# Possible Regulatory Roles of Promoter G-Quadruplexes in Cardiac Function-Related Genes – Human *TnIc* as a Model

**DOI:** 10.1371/journal.pone.0053137

**Published:** 2013-01-09

**Authors:** Wenhua Zhou, Kogularamanan Suntharalingam, Nigel J. Brand, Paul J. R. Barton, Ramon Vilar, Liming Ying

**Affiliations:** 1 Molecular Medicine, National Heart and Lung Institute, Imperial College London, London, United Kingdom; 2 Department of Chemistry, Imperial College London, London, United Kingdom; 3 Harefield Heart Science Centre, National Heart and Lung Institute, Imperial College London, Middlesex, United Kingdom; 4 NIHR Cardiovascular Biomedical Research Unit, Royal Brompton and Harefield NHS Trust, London, United Kingdom; Universite de Sherbrooke, Canada

## Abstract

G-quadruplexes (G4s) are four-stranded DNA secondary structures, which are involved in a diverse range of biological processes. Although the anti-cancer potential of G4s in oncogene promoters has been thoroughly investigated, the functions of promoter G4s in non-cancer-related genes are not well understood. We have explored the possible regulatory roles of promoter G4s in cardiac function-related genes using both computational and a wide range of experimental approaches. According to our bioinformatics results, it was found that potential G4-forming sequences are particularly enriched in the transcription regulatory regions (TRRs) of cardiac function-related genes. Subsequently, the promoter of human cardiac troponin I (*TnIc*) was chosen as a model, and G4s found in this region were subjected to biophysical characterisations. The chromosome 19 specific minisatellite G4 sequence (MNSG4) and near transcription start site (TSS) G4 sequence (−80 G4) adopt anti-parallel and parallel structures respectively in 100 mM KCl, with stabilities comparable to those of oncogene G4s. It was also found that TnIc G4s act cooperatively as enhancers in gene expression regulation in HEK293 cells, when stabilised by a synthetic G4-binding ligand. This study provides the first evidence of the biological significance of promoter G4s in cardiac function-related genes. The feasibility of using a single ligand to target multiple G4s in a particular gene has also been discussed.

## Introduction

Although the majority of human genomic DNA adopts the iconic double helical structure (also known as B-form or duplex DNA) proposed by Watson and Crick [1], other non-B-form DNA secondary structures formed within certain sequences in the human genome have also been revealed in many *in vivo* and *in vitro* studies, such as the G-quadruplex DNA (G4 DNA) and i-motifs (i-tetraplexes) formed in oligonucleotides with guanine and cytosine tracts respectively (reviewed in [2], [3]). As 98% of the human genome is non-coding, the possible biological functions of these transient and dynamic non-B-form DNA structures have been a subject of increasing interest.

As compared to other non-B-form DNA, G4s are relatively stable in solution under near-physiological conditions (thermodynamic and kinetic stability of G4s are reviewed in [4] and [5], respectively), which enables them to compete with adjacent duplex DNA and therefore participate in certain biological processes. The accumulated distribution of G4s in gene promoter regions throughout the human genome also makes them particularly important as compared to other non-B-form DNA structures [6]. Unsurprisingly, there have been a growing number of studies on the biological functions of these DNA structures, since the discovery that G4s can form in the G-rich regions from human telomeric oligonucleotides in the late 1980s [7].

Early studies about the regulatory roles of promoter G4s have focused on a few specific gene loci. For example, the formation of G4 structures (mostly stabilised by certain G4-binding ligands) in promoters of the human insulin [8], [9], *MYC* [10], [11], *KRAS* [12], [13], and *PDGF-A* [14] genes were shown to have significant influences on downstream gene transcription activity. Subsequently, promoter G4s have been identified in important functional regions (promoters, NHE regions, enhancers, and so on) of many other cancer-related genes, and their biophysical properties in solution have been thoroughly characterised. Examples include the G4s found in promoters of *KIT* [15]–[19], *RET* [20], *VEGF* [21]–[24], *RB1* [25]–[27], *BCL-2* [28]–[30], and *HIF-1α* [31] genes. These isolated examples have led to the hypothesis that G4s may act as a group of common *cis*-regulatory elements via various mechanisms *in vitro* and *in vivo*. Indeed, accumulating bioinformatics studies in the past decade supported this hypothesis as described below.

Firstly, the enrichment and biased distribution of G4s are found in gene transcription regulatory regions in the human genome, such as in promoter regions, 5′-ends of 5′-UTRs, within the first intron and within regions immediately downstream of the 3′-ends of genes. These findings indicate that these non-canonical DNA structures are favoured by natural selection in the process of evolution. Secondly, potential G4-forming motifs closely correlate with functional regions in gene promoters in the human genome, such as the gene regulatory elements [32] and G-rich TF (transcription factor) binding sites [33], [34], which further supports the hypothesis that G4s are involved in gene transcription regulation. Furthermore, the existence of potential G4-forming motifs is also related with downstream gene expression levels, indirectly demonstrating the regulatory roles of G4s in gene transcription regulatory regions (TRRs) [35], [36]. Finally, correlation between prevalence of G4s and gene functions also indicates potential roles of G4s in the human genome [37].

Due to its diverse biological functions and wide distribution in the human genome, G4 DNA has attracted exceptional attention from nucleic acid chemists to design and synthesise novel G4-binding ligands that can interact efficiently and selectively with this peculiar DNA structure. So far, most G4-binding ligands are researched as potential anti-cancer agents by targeting cancer-related G4s, especially the human telomeric G4 and oncogene promoter G4s. The affinity and selectivity of G4-binding ligands to G4s rather than to other DNA forms are mainly achieved by π–π stacking and electrostatic interaction, which are also the two main criteria in G4-binding ligand design [38], [39].

To date, almost all G4 studies have focussed on their involvement in cancer and therefore their potential as targets for anticancer agents. Thus, most studies have centred on telomeric G4 and promoter G4s in proto-oncogenes. Importantly, extensive studies of these cancer-related G4s have contributed greatly to forward our knowledge in this field, which have established the fundamentals to investigate the role of G4s in other genes, such as those involved in regulating cardiac functions. Since possible biological functions of G4s have been identified in some heart-related genes, such as *PDGF-A*, *VEGF-A* and *HIF-1α*, G4 research also has relevance to cardiovascular science.

Taking into account the instability of G4s and their much lower abundance as compared to adjacent duplex DNA in the human genome *in vivo*, and the structural similarity between different G4s, it makes the design of selective G4-binding ligands extremely challenging, and off-target effects are practically inevitable. However off-target effects may lead to multi-targeting effects in some cases, which in turn become advantageous for G4-binding ligands, and may enhance their therapeutic potential, such as the ligand naphthalene diimide derivative 1 [40], 12459 [41]–[43], and CX-3543 [44].

In this article, we first evaluated, by bioinformatics approaches, the importance of G4s in TRRs of key cardiac genes as compared to other genes, especially those involved in cancer. We found that potential G4-forming motifs are particularly enriched in TRRs of genes active in the heart, and TRRs of genes involved in cardiac function-related pathways are also highly G4-abundant. Although very basic, these findings clearly supported the hypothesis that G4s are important in regulating cardiac function-related genes. Furthermore, due to the abundance of G4-forming motifs in TRRs of cardiac function-related genes, potentially, multi-targeting strategy may have similar therapeutic potential as those observed in anti-cancer research.

To validate the bioinformatics results (i.e. that G4s are involved in regulating cardiac function-related genes) and to initially test the multi-targeting strategy within a single gene, subsequently, the human cardiac troponin I (*TnIc*) gene was selected as a model system. Human TnIc is the only sarcomeric protein found to be exclusively expressed in the cardiac myocyte, which makes it an ideal model to investigate cardiac-specific gene expression and in turn to provide new approaches to the construction of cardiac-specific vectors in gene therapy. Although TnIc has been regarded as a preferred biomarker in MI (myocardial infarction) diagnosis for more than a decade [45], [46], the molecular mechanisms of human *TnIc* transcription regulation are still poorly understood, and only a few TFs have been found to be involved in regulating the transcription of *TnIc* [47]–[49]. This is partially due to the difficulties in obtaining suitable cell models, and possibly due to the extremely G-rich in its promoter. Indeed, TRR of *TnIc* is highly G4-enriched according to the bioinformatics analysis. Multiple G4-forming sequences have been identified in the proximal promoter of human *TnIc*, and two types of G4-forming motifs were selected for detailed studies, which are designated as “MNSG4” (between −528 and −319 bp upstream to the TSS, transcription start site), and “−80 G4” (centred at −80 bp to the TSS). The MNSG4 motif is composed of 6 repeats of chromosome 19 specific G-rich minisatellite (MNS), which are highly conserved in primates. Although conserved chromosome 19 specific MNS repeats are also found in many other genes, such as the neuronally expressed Shc adaptor homolog *SCK1/SLI* [50], the EFG-like module-containing mucin-like receptor, *EMR3* [51], the human apolipoprotein C-II gene [52], and the human *TnT* [53], the actual functions of this widely distributed G4-forming sequence are still unclear. On the other hand, similar as the KIT1 G4-forming sequence, the −80 G4 is found to be unique in the human genome [18]. More importantly, multiple putative or proven TF binding sites are clustered within or around the −80 G4-forming region, such as a CACC-box, an Sp1 binding site, an E-box, and a GATA biding site [48].

We have used a combination of biophysical and biochemical approaches to characterise the formation, stability and possible regulatory roles of the G4s (MNSG4 and −80 G4) in the promoter of the human *TnIc*. The methods include CD (circular dichroism) spectroscopy [3], EMSA (electrophoresis mobility shift assay) [7], FRET (fluorescence resonance energy transfer) [54], DMS (dimethyl sulfate) footprinting [31], FID (fluorescent intercalator displacement) experiments [55], and dual luciferase reporter assays [10] in HEK293 cell line. These methods are reviewed in [56] and described in detail in [57]. It was found that these G4s have comparable stability as that of proto-oncogene G4s, and they may act cooperatively as enhancers in gene expression regulation in HEK293 cells when they are stabilised by a synthetic G4-binding ligand. This study provides the first evidence about the biological significance of cardiac promoter G4s.

## Materials and Methods

### Enrichment of G4-forming Motifs in Different Tissues

Sequences of the human genome (version 37.64) were downloaded from ENSEMBL FTP, and gene/transcripts features were exported from BioMart (detailed protocols are described in Protocols S1). TRR of individual transcripts were defined as −2,000 bp to +1,000 bp from TSS of the transcript. Although the actual mechanisms and final consequences of downstream and upstream (relative to TSS) G4s are different, we are only interested in whether or not G4s are involved in regulating a particular gene expression, and thus down- and upstream G4s are investigated together in most cases in this study. G4-forming motifs were identified as G_≥3_(N_1∼7_G_≥3_)_≥3_. Tissue-specific enrichment of G4-forming motifs was defined as the percentage of genes active in certain tissue (GNF/Atlas organism part) containing at least one G4-forming motif in a certain region of the TRR (Equation 1)
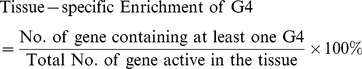
(1)


Besides the full TRRs, enrichment of G4s was also analysed and compared in the distal promoter region (−2,000∼−501 bp), proximal promoter region (−500∼−1 bp), and downstream region (TSS∼+1,000 bp). The analysis was carried out in 10 iterations to consider transcript redundancy. Hence the tissue-specific enrichment of G4-forming motifs was presented as mean ± standard deviation (SD). In analysing specific tissue, differences among iterations were evaluated by one-way ANOVA test, in which *P*≥0.05 represents that no significant difference was found.

### Importance of G4s in Different Pathways

To evaluate the importance of G4s in individual transcript, two scores, *F* and *Q*, were calculated respectively to reflect the abundance and location significance of G4s identified in each transcript. In the TRR of each transcript, the percentage of nucleotides involved in G4 formation is defined as *F* values according to Equation 2

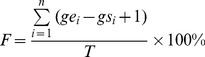
(2)where *n* is the number of G4s identified in the TRR (*F* = 0 when *n* = 0), *gs_i_* and *ge_i_* are the start and end positions of *i* ^th^ G4 identified in the TRR relative to the TSS in 5′ to 3′ direction, and *T*, the length of TRR being searched, is fixed at 3,000 in this study.

Abundance of G4s on the coding strand, and template strand were separately calculated and labelled as *F*_cd_, and *F*_ncd_, (subscripts cd: coding strand; ncd: noncoding strand). Overall abundance of G4s, the *P*_o_ (o: overall), were calculated as the sum of *F*_cd_, and *F*_ncd_. To genome-widely compare the abundance of G4s in TRRs of different transcripts, *F* scores of all transcripts without redundancy were calculated (one transcript was randomly picked when multiple transcripts were reported from the same gene), and the cumulative frequencies of *F* scores of all transcript were calculated and denoted as *CF*_cd_, *CF*_ncd_, and *CF*_o_. To reach a more stringent criterion, transcripts with *F* scores equal to 0 were excluded when calculating *CF*. Genes with transcript *CF* values higher than 50% were regarded as G4-rich in their TRR, while those with transcript *CF* values lower than 50% were regarded as G4-scarce.

Except for the G4 abundance, the location of individual G4 in a particular TRR may also correlate with its biological significance. As reported in previous genome-wide analyses, G4 was found to be enriched in the promoter region between −200 bp and TSS, and peaked at around −50 bp to TSS in the human genome [58]. This positional bias of G4 distribution in human gene promoters is believed to be a result of evolutionary pressure [59]. Most promoter G4s with confirmed biological functions localize in this region, such as *KIT* and *MYC* promoter G4s. Thus, another indicator, the *Q* score, is introduced to evaluate the potential location significance of G4s in particular TRR.

Following on the previously reported method [58], the probability distribution of each TRR position relative to TSS involved in G4 formation along coding and template (noncoding) strands of all TRRs was calculated according to Equation 3

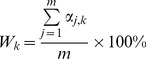
(3)where *W_k_* is the normalized probability of nucleotide in position *k* of all TRRs involved in G4 formation, *m* is the total number of transcripts analyzed. *α_j,k_* is the logical value of nucleotide in position *k* of *j* ^th^ TRR; *a_j,k_* = 1 when the nucleotide is involved in G4 formation, otherwise *a_j,k_* = 0. G4 location significance score *Q* (*Q*_cd_, *Q*_ncd_ and *Q*_o_ for coding and template strand, and overall, respectively; *Q*_o_ = *Q*_cd_+*Q*_ncd_) for the TRR of a particular transcript was calculated according to Equation 4



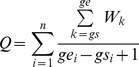
(4)The cumulative frequencies of *Q* scores, *CQ,* were also calculated. Similarly, transcripts with the zero *Q* scores were excluded. Genes with both *CF* and *CQ* values higher than 50% were regarded as G4-important genes, while those with both values lower than 50% were regarded as G4-less-important.

Correlation between the *CF* and *CQ* scores on the coding, template strand and both strands were also investigated. The correlation coefficient was calculated according to Equation 5

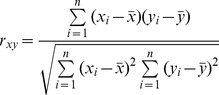
(5)in which *x*_i_ and *y*_i_ are the *CF* and *CQ* scores of the coding, template strand or both strands at genome level, 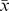 and 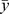 are the corresponding mean values *CF* and *CQ* scores at genome level.

*CF* and *CQ* were calculated for all transcripts without redundancy. To evaluate bias introduced in random-picking when multiple transcripts exist in one gene, calculation was carried out for 10 iterations. Gene list for all pathways was downloaded from KEGG database. All pathways including more than 50 genes were subject to pathway analysis. When more than 50% component genes in a pathway were calculated as G4-important (excluding those with both *CF* and *CQ* equal to zero), the pathway was regarded as G4-important. To evaluate the G4-importance of individual pathway, distribution of *CF* and *CQ* values of pathways were compared to the distribution of all transcripts in humane genome by Wilcoxon rank sum test. Differences between iterations of individual pathway were evaluated by one-way ANOVA test.

The source code of bioinformatics studies is available upon request.

### DNA Oligos

All labelled and unlabelled DNA oligos used in biophysical studies were purchased from IBA Biotechnology (Göttingon, Germany) and HPLC purified (double HPLC purification for labelled oligos). Fluorophores (Cy3 or Cy5) were coupled to nucleotides via NHS (N-hydroxysuccinimide) esters. Oligo concentration was determined by absorption at 260 nm. Oligo **Tr_MNS_-I** is the consensus sequence of G4-forming minisatellites between −528 and −319 bp to the TSS of human *TnIc* gene. Oligo **Tr_MNS_-II** is the C-rich complimentary strand of oligo **Tr_MNS_-I**. Oligo **Tr_MNS_-III** is the Cy3-labeled **Tr_MNS_-I** attached with a 34-mer artificial linker on its 3′-end. **Tr_-80_-I** is a G4-forming sequence from the noncoding strand fragment of human *TnIc* promoter localized between −97 and −73 bp to the TSS, while oligo **Tr_-80_-II** is its C-rich complimentary strand. Oligo **Tr_-80_-III** is the Cy3-labeled **Tr_-80_-I** attached with a 34-mer artificial linker on its 3′-end. Oligo **Comp-Cy5** is a Cy5-labeled oligo complimentary to the 34-mer artificial linker in oligos **Tr_MNS_-III** and **Tr_-80_-III**. Oligos and primers used in plasmid construction were purchased from Eurofins MWG Operon (Ebersberg, Germany) and were subjected to HPLC purification. The sequences and labeling sites of these oligos are listed in Table 1.

**Table 1:** Details of oligos used in biophysical studies in solution.

Name	Sequence (5′–3′)
*Synthetic oligos used in biophysical studies*	
Tr_MNS_-I	TGG GTC TGA GGG AGG AGG GGC TGG GGG C
Tr_MNS_-II	GCC CCC AGC CCC TCC TCC CTC AGA CCC A
Tr_MNS_-III	^Cy3^TGG GTC TGA GGG AGG AGG GGC TGG GGG CGA GGT AAA AGG ATA ATG GCT ACG GTG CGG ACG GC
Tr_MNS_-IV	^Cy3^TGG GTC TGA GGG AGG AGG GGC TGG GGG ^Cy5^C
Tr_-80_-I	CGG GGG CGC GTG AGG GGC GGG GTG GGC
Tr_-80_-II	GCC CAC CCC GCC CCT CAC GCG CCC CCG
Tr_-80_-III	^Cy3^CGG GGG CGC GTG AGG GGC GGG GTG GGC GAG GTA AAA GGA TAA TGG CTA CGG TGC GGA CGG C
Tr_-80_-IV	^Cy3^CGG GGG CGC GTG AGG GGC GGG GTG GG ^ Cy5^C
Comp-Cy5	GCC GTC CGC ACC GTA GCC ATT ATC CTT ^Cy5^TTA CCT C
*Synthetic oligos used in plasmid construction*	
MNS_G	CCT GGA CTC TTG GGT CTG AGG GAG GAG GGG CTG GGG G
MNS_C	TCC AGG CCC CCA GCC CCT CCT CCC TCA GAC CCA AGA G
MNSM_G	CCT GGA CTC TTG AAT CTG AGA GAG GAG AGA CTG AGA G
MNSM_C	TCC AGG CTC TCA GTC TCT CCT CTC TCA GAT TCA AGA G
5′Linker_G	GGG GAC AAG TTT GTA CAA AAA AGC AGG CTG AAT TC
5′Linker_C	TCC AGG GAA TTC AGC CTG CTT TTT TGT ACA AAC TTG TCC CC
3′Linker_G	CCT GGA CAC TTG AGT CTG CAG ACC CAG CTT TCT TGT ACA AAG TGG TCC CC
3′Linker_C	GGG GAC CAC TTT GTA CAA GAA AGC TGG GTC TGC AGA CTCAAG TG

Note: ^Cy3/5^N indicates a Cy3 or Cy5-labeled nucleotide, which is coupled via NHS ester.

### Formation of G4s in Solution

Formation of G4s was determined by EMSA, CD spectroscopy, and DMS footprinting in solution. Detailed protocols are described in Protocols S1.

### G4 Unfolding Thermodynamics and Kinetics

Unfolding thermodynamics of G4s and the stabilising ability of G4-binding compounds to these G4s were investigated by CD melting experiments. Unfolding kinetics of these G4s were investigated via a pseudo-first order approach as described previously [60]. Briefly, the fluorophores-labeled unfolded G4s were trapped by excess C-rich oligos, and hence resulted in decreased FRET signals. Detailed protocols are described in Protocols S1.

### G4-ligand Interaction in Solution by FID (Fluorescent Intercalator Displacement) Assay

The corresponding oligonucleotides were first dissolved in MilliQ water to yield a 20 µM stock solution, and then were diluted in 10 mM potassium cacodylate (pH 7.4)/50 mM potassium chloride (60 mM K^+^) buffer to the appropriate concentrations. Prior to use in the FID assay, the DNA strands were incubated to allow the formation of G4s as stated above. The compounds to be analysed and thiazole orange (TO) were dissolved in DMSO to give 1 mM stock solutions. The corresponding solution was then diluted using 10 mM potassium cacodylate (pH 7.4)/50 mM potassium chloride (60 mM K^+^) buffer to the appropriate concentrations. The FID assay was carried out according to the protocol as previously reported [55]. Briefly, to a mixture of DNA sequence (0.25 µM) and TO (0.50 µM) in 10 mM potassium cacodylate (pH 7.4)/50 mM potassium chloride (60 mM K^+^) buffer an increasing amount of the molecule under study was added (0.125 to 5 µM, which corresponds to 0.5 to 20 equiv). After an equilibration time of 3 min the emission spectrum was recorded between 510 and 750 nm with an excitation wavelength of 501 nm. This was recorded using a Varian Cary Eclipse Spectrometer (Agilent Technologies, Yarnton, UK). The fluorescence area was calculated using the “trapezium rule” method. The area was converted into percentage TO displacement by the following formula: % TO displacement = 100 − [(fluorescence area of sample/fluorescence area of standard) × 100]. The standard fluorescence spectrum was obtained in the absence of any G4-binding ligands. % TO displacement was then plotted against each of the compound concentrations to give the respective FID curves, from which the DC_50_ values were determined.

### Construction of Wild-type/Mutated Minisatellite Concatemer

Engineered wild-type (E-WT, oligo **MNS_G/C**) and mutated (M, oligo **MNSM_G/C**) minisatellite concatemers were constructed by ligation and recombination processes. Oligos **MNS_G/C** and **MNSM_G/C** were first phosphorylated individually in 10 µl phosphorylation reaction system (DNA 250 pmol; 10× PNK buffer, 1 µl; 10 mM ATP, 1 µl; 10 U/µl PNK, 0.5 µl) at 37°C for 1 hour. Then, phosphorylated oligos **MNS_G/C** and **MNSM_G/C** were annealed to each other by a PCR thermocycler (Stratagene) at 95°C for 5 min, then gradually cooling to 25°C over 2 hours. Concatemers with random number of repeat were constructed by self-ligation (first round ligation) of annealed single repeat of E-WT/M minisatellites. 5′linker and 3′linker were phosphorylated and annealed from oligos **5′Linker_G/C** and **3′Linker_G/C** respectively through the same protocol. Then, annealed 5′linker was ligated to concatemers in a second round ligation, and the 3′linker was added in the third ligation subsequently. All three rounds ligation were carried out in 20 µl system at 16°C for 2 hours. Ligation products larger than 100 bp were extracted by QIAGEN Gel extraction kit after each round of ligation. All enzymes and buffers used in this step were purchased from NEB (Hitchin, UK).

### Construction of Luciferase Vector

**Figure 1:**
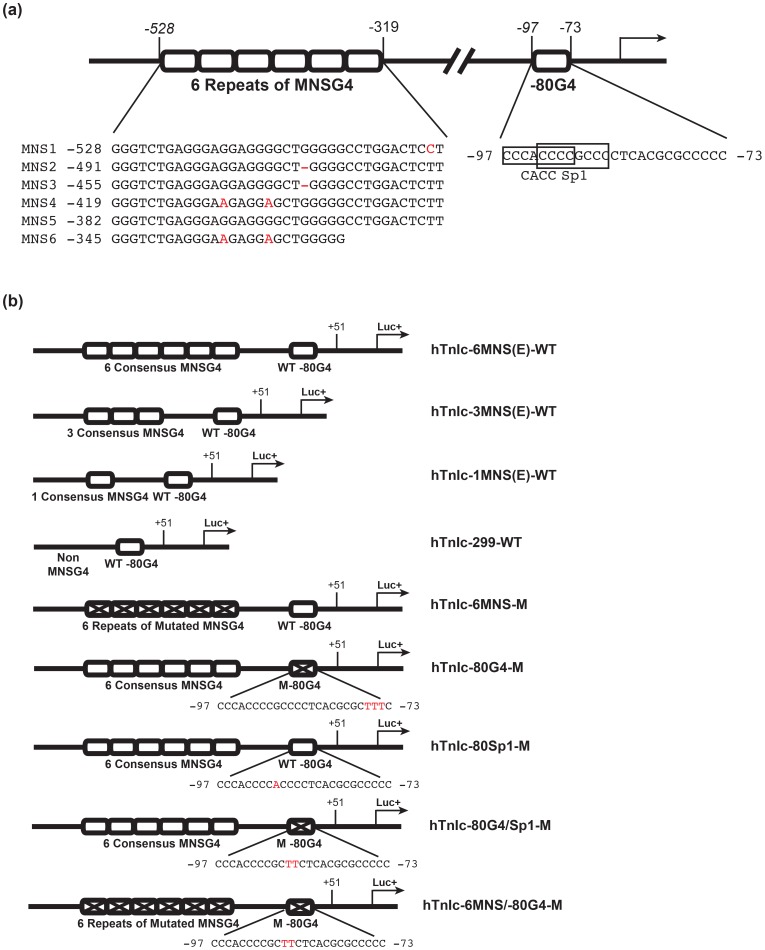
Structure of human TnIc gene promoter and constructed luciferase expression vectors. (**a**) Schematic illustration of the G4-forming motifs in the promoter of human *TnIc* gene. (**b**) the structure of luciferase expression vectors driven by engineered human *TnIc* promoters.

Entry vector containing minisatellite concatemers was made by recombinating the third round ligation products into pDONR221 using Gateway® BP reaction kit (Invitrogen). The minisatellite concatemers were further recombinated into a pGL3-Basic-Gateway vector using Gateway® LR reaction kit (Invitrogen). The insertions were confirmed by sequencing using M13 primer pair. The −299 to +51 fragment of human *TnIc* promoter was PCR amplified from plasmid pGL3-Basic_−553 containing the −553 to +69 fragment of human *TnIc*. Plasmid pGL3-Basic_−553 is a kind gift from Dr. Nigel Brand. Vectors hTnIc-6MNS(E)-WT, hTnIc-3MNS(E)-WT, and hTnIc-1MNS(E)-WT plasmids were constructed by inserting the −299 to +51 fragment into pGL3-Basic-Gateway vectors downstream to the engineered WT minisatellite concatemers with 6 repeats, 3 repeats, and 1 repeat via *Pst*I and *Xho*I restriction sites. By using similar approach, the vector containing 6 repeats of mutated MNSG4 was constructed as hTnIc-6MNS-M. Vector hTnIc-299-WT was constructed by inserting the −299 to +51 fragment into empty pGL3-Basic-Gateway vector via *EcoR*I and *Pst*I restriction sites. Vectors hTnIc-80 G4-M, hTnIc-80Sp1-M, and hTnIc-80 G4/Sp1-M were derived from hTnIc-6MNSG(E)-WT by *Dpn*I-mediated site-direct mutagenesis, in which the −80 G4-forming sequence, the Sp1 binding site in the −80 G4 motif, and both of them were mutated respectively. An overall mutant hTnIc-6MNS/80 G4-M was generated from hTnIc-6MNS-M by mutating both −80 G4-forming sequence and the Sp1 binding site in it. Vectors are schematically illustrated in Figure 1.

### Transfection of Cultured Human Cells and G4-binding Ligands Treatment

HEK293 cells were inoculated in 75 cm^2^ flasks and grown to 70–80% confluence at 37°C and under 5% CO_2_ atmosphere in complete medium (Dulbecco Modified Eagle’s Medium (DMEM) supplemented with 4500 mg/L D-glucose, 5.0 mM L-glutamine, 10% fetal calf serum, and 83.3 U/ml of penicillin and streptomycin (Invitrogen)). The cells were harvested by Trypsin-EDTA, and reseeded 2×10^4^ cells/well in 24 well plates with a volume of 300 µl/well complete medium, and the cells were cultured overnight. The medium was replaced by 300 µl fresh medium at least 2 hours before transfection. Transfection was carried out by following calcium phosphate precipitation. Briefly, 20 ng pRL-TK control plasmid (Renilla luciferase reporter vector driven by herpes simplex virus thymidine kinase promoter P_HSV-TK_) and 150 ng testing pGL3 luciferase plasmid was transfected in each well, and cells were incubated with precipitated DNA for another 24 hours before adding G4-binding ligands. G4-binding ligands were diluted in DMSO to different concentrations as 1000× stock solution. 0.3 µl G4-binding ligand stock solution was diluted to 300 µl by fresh medium to replace the transfection medium in each well. Cells were then incubated with G4-binding ligands for 24 hours before cell lysis for luciferase assays. Experiments were carried out in triplicates.

### Luciferase Assay

Transfected cells were firstly washed by ice-cold PBS to reduce background signals from the medium, and luciferase assays were performed subsequently according to manufacturer’s instruction (Promega). Generally, cells were lysed by 100 µl passive lysis buffer per well, and 10 µl cell lysate were added to 50 µl luciferase assay reagent. After 3 s’ delay, luciferase signals were collected for 10 s in a Glomax 20/20 luminometer (Promega). After adding 50 µl of Stop and Glo reagent, Renilla signals were collected for 10 s as internal control.

## Results

### Enrichment of G4 DNA in the Heart

The enrichment of G4s in the transcriptional regulatory regions (TRRs: −2,000 bp to +1,000 bp around Ensembl Transcript Start) of all known protein-coding genes was first analysed as a reference point. In total, of 19,633 genes that were analysed, 67.79±0.01% of them were found to contain at least one G4 on both coding strand and template strand, with 52.42±0.02% and 46.91±0.02% containing at least one G4 on the coding strand (G4) and template strand (C4), respectively. Compared with the widely accepted results that around 40% human gene promoters contain G4s [6], the much higher percentage (67.79±0.01%) identified here can be attributed to the much longer region around the TSS that was analysed in this study. Similar results have been previously reported in the chicken genome when regions of similar length were analysed [61].

**Figure 2:**
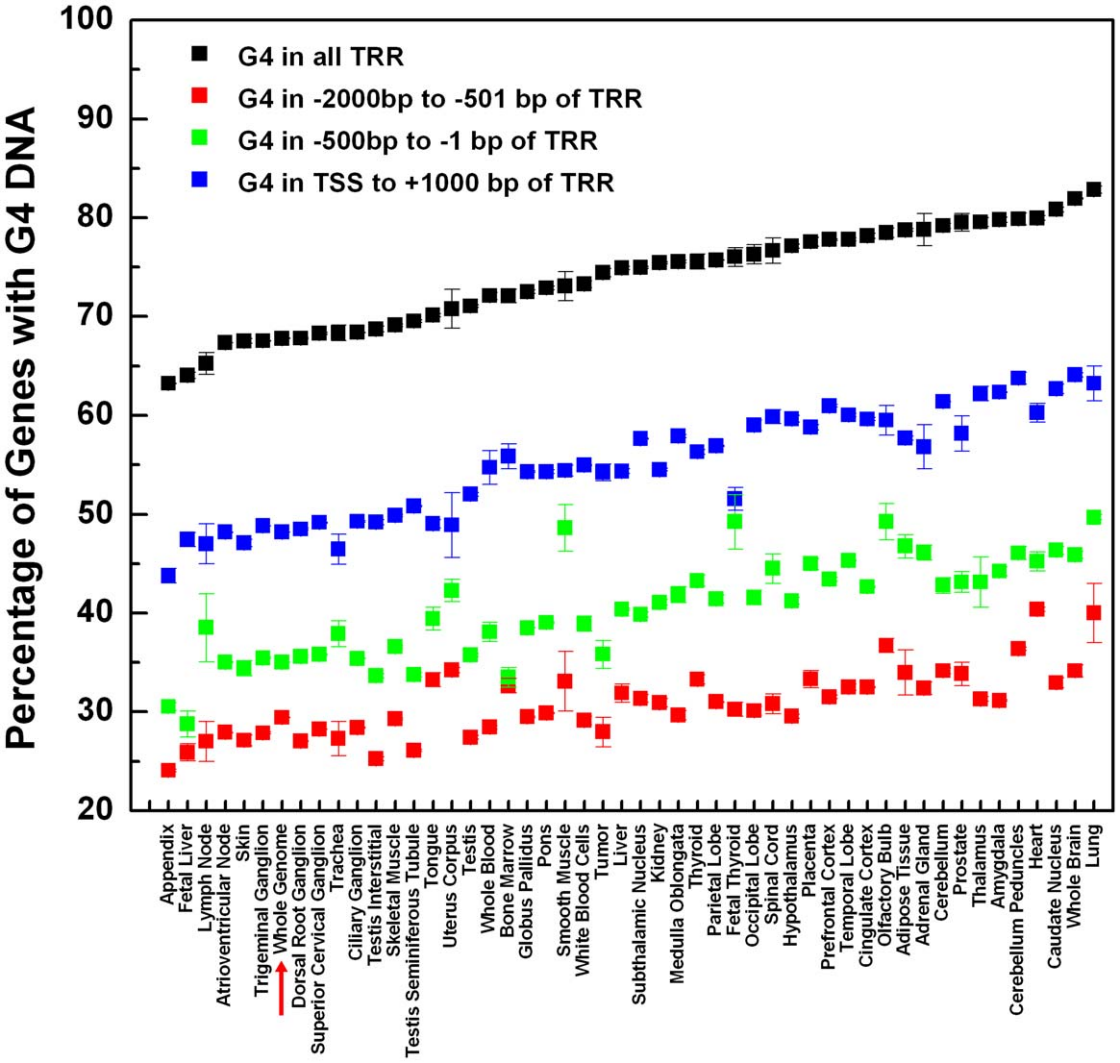
Enrichment of G4s in TRRs of genes active in different tissues. The percentage of genes active in different tissues containing at least one G4-forming sequence in the whole TRR (black), −2,000∼−501 bp of TRR (red), −500∼−1 bp of TRR (green), and TSS∼+1,000 bp of TRR (blue). Enrichment of G4s in all protein-coding genes (G4 enrichment at genome level) is indicated by the red arrow. The calculation was repeated randomly for ten times (multiple transcripts of one gene were randomly chosen for ten times) to take into account the redundancy effects of multiple transcripts.

The enrichment of conserved G4s in different tissues has been previously investigated by Verma *et al*. [35]. Following a similar method, we focused on investigating the enrichment of G4s in different regions of gene TRR in different tissues (different GNF/Atlas organism) in the human genome. As shown in Figure 2 and Table S1, G4s are highly enriched in the whole TRR of genes active in the lung, heart and brain including cerebellum peduncles and caudate nucleus. The high G4 enrichment in these tissues may indicate that these tissues have evolved certain mechanisms to utilize G4s to regulate gene transcription (i.e. providing binding sites for tissue-restricted TFs, maintain the separation of coding and template strands, and so on), or at least to avoid possible negative effects caused by the formation of G4s in TRR (i.e. to resolve folding of G4 by helicases). For tissues with the lowest G4 enrichment at the whole TRR level, corresponding G4 enrichment in different regions of TRR is also among the lowest. Interestingly, for some tissues, although moderate or relatively low G4 enrichment was found at whole TRR level, G4s were found to be enriched in either distal or proximal promoters in the TRRs. For example, in smooth muscle, although the overall G4 enrichment is only moderately higher than that found at the whole genome level, its G4 enrichment in the proximal promoter region is among the highest. Similar phenomena were found in tissues of adipose, olfactory bulb, and fetal thyroid (Table S1). This may indicate that in these tissues certain mechanism may exist to utilize G4s in the proximal promoter region, but not in other regions.

According to the above findings, several points became apparent. Firstly, enrichment of G4 in TRR of genes has a strong tissue-specificity. Secondly, in some tissues (smooth muscle, adipose tissue, olfactory bulb, and fetal thyroid), G4s are enriched specifically in certain regions of TRRs (location-specificity) reflecting possible tissue-related regulatory roles of G4s. These tissues, with either tissue- or location-specific enrichment of G4s in TRRs, are worthy of further investigation in the future.

From the studies described here, G4s appear to be highly abundant in TRRs of genes active in the cardiac function-related tissues, such as the heart and smooth muscle. To further investigate the biological significance of G4s in the heart, we analysed the abundance and location significance of G4s in TRRs of genes involved in different pathways described within the KEGG (Kyoto Encyclopedia of Genes and Genomes) PATHWAY database, especially those related to cardiac functions. The importance of G4s in each TRR was evaluated by two scores: G4 abundance score (*F* score, and its cumulative frequency: *CF*) and G4 location significance score (*Q* score, and its cumulative frequency *CQ*), which will be described separately in following sections.

### *F* Score

As compared to duplex DNA, G4s are normally less stable (reviewed in [4], [62], [63]). Thus, high G4 content (abundance) in a particular TRR can enhance the competition of G4 DNA with adjacent duplex DNA, resulting in the increased probability of the involvement of G4s in regulating downstream gene expression. Based on this assumption, the *F* score representing the abundance of G4s in a particular TRR was used as the first indicator to evaluate the importance of G4 in the TRR of each gene (methods modified from Eddy *et al.* [37]). For each transcript, the *F* score was calculated based on the percentage of nucleotides involved in G4 formation in the TRR of that transcript (Equation 2). By including all transcripts with redundancy, the coding strand, template strand (non-coding strand), and both strands were analysed separately to give the *F*_cd_, *F*_ncd_, and *F*_o_ scores, respectively (Table 2). Figure S1 shows the distribution of these *F* scores obtained by including all transcripts with redundancy. A similar skewed distribution of G4 abundance was reported previously in an analysis showing G4 formation potential (G4P) in RefSeq of genes [37]. However, due to the higher resolution in this study (using the percentage of nucleotides involved rather than the percentage of a shifting searching window containing G4-forming motifs), two peaks were found in the distribution of G4 abundance scores. By comparing the peak position and corresponding values in the distribution histograms of G4 abundance on coding and template strand (Figure S1 a and b, lower graphs), G4-forming motifs are more enriched in the coding strand. All values were re-calculated with 10 iterations with randomly chosen transcripts, and compared with values from transcripts with redundancy, and similar results were obtained (Table 2). The *CF*_cd_, *CF*_ncd_, and *CF*_o_ values for each transcript were calculated as the cumulative frequency according to the *F*_cd_, *F*_ncd_, and *F*_o_ histograms (Figure S1, upper graphs).

**Table 2:** Comparison of G4 abundance in TRRs of transcripts (the *F* score).

	Median Value	Mean Value	Max Value
*Transcripts with Redundancy (46,205 Transcripts)*			
***F*** **_cd_**	1.6	2.1	33.2
***F*** **_ncd_**	1.4	1.9	29.6
***F*** **_o_**	2.2	2.9	33.2
*Transcripts without Redundancy (19,633 Transcripts), 10 iterations* *			
***F*** **_cd_**	1.55±0.02	2.03±0.01	33.2
***F*** **_ncd_**	1.42±0.02	1.93±0.01	25.88±3.96
***F*** **_o_**	2.13±0.01	2.90±0.01	33.2

* The *F*_cd_, *F*_ncd_, and *F*_o_ values from 1^st^ iteration were used as templates and compared with those from following iterations by one-way ANOVA test. No differences among iterations were observed (*P*≥0.05).

### *Q* Score

Besides the abundance, the locations of G4s in a particular TRR are also important in estimating the potential biological significance of G4. For example, Du *et al.* suggested that the colonization of G4-forming motifs with TF binding sites in the proximal promoters indicates potential regulatory roles of G4s [32]. Thus, we believe that the biased probability distribution of G4-forming motifs in TRRs at whole genome level, which was reported by Huppert *et al.* [58], may reflect the preference of G4 location in TRRs through evolution. Following Equation 3 (modified from Huppert *et al.* [58]), the probability distribution histogram was generated (Figure S2 a). Similar probability distribution of G4 around TSS has been reported in the genomes of other warm-blooded animals [59]. The location and strand polarity-dependent distribution of G4s appears to reflect an evolutionary pressure, and it is necessary to take into account the location of G4 motifs when considering their importance in the TRR of a particular gene. Therefore, a location significance score *Q* was introduced in order to characterise the location significance of G4s. For each TRR, its G4 location significance scores on the coding (*Q*_cd_) and template (*Q*_ncd_) strand were calculated according to Equations 3 and 4. The overall G4 location significance score (*Q*_o_) was determined as the sum of *Q*_cd_ and *Q*_ncd_. Histograms *Q*_cd_, *Q*_ncd_, and *Q*_o_ for TRRs of all transcripts with redundancy are shown in Figure S2 b∼d (lower graphs). The larger maximum, median, and average *Q* scores on the coding strand also reflect the higher location significance of G4s on the coding strand as compared to the template strand. Transcripts without redundancy were also analysed with 10 iterations of randomly chosen transcripts, and similar results were obtained as compared to the results from transcripts with redundancy (Table 3). The *CQ* values for each transcript were calculated as its cumulative frequency in *Q* histogram and are shown in Figure S2 b∼d (upper graphs).

**Table 3:** Comparison of G4 location significance in TRRs of transcripts (the *Q* score).

	Median Value	Mean Value	Max Value
*Transcripts with Redundancy (46,205 Transcripts)*			
***Q*** **_cd_**	2.5	3.3	26.9
***Q*** **_ncd_**	1.7	2.3	19.8
***Q*** **_o_**	3.2	4.2	29.3
*Transcripts without Redundancy (19,633 Transcripts), 10 iterations* *			
***Q*** **_cd_**	2.48±0.02	3.13±0.02	23.4±1.6
***Q*** **_ncd_**	1.85±0.02	2.40±0.02	19.7±0.2
***Q*** **_o_**	3.20±0.02	4.07±0.01	28.3±0.2

* The *Q*_cd_, *Q*_ncd_, and *Q*_o_ values from 1^st^ iteration were used as templates and compared with those from following iterations by one-way ANOVA test. No differences among iterations were observed (*P*>0.23).

### Correlation between G4 Abundance and G4 Location Scores

**Figure 3:**
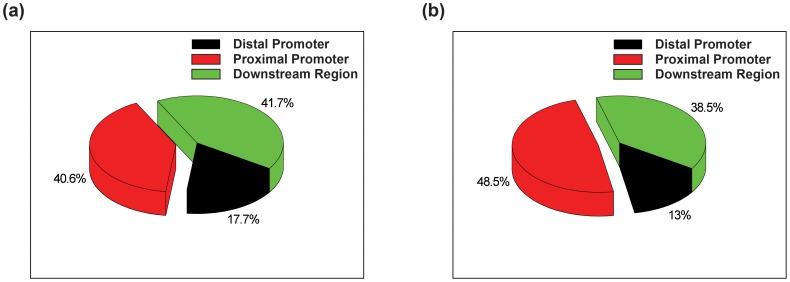
The normalised percentage of G4s in different regions of TRRs of G4-less-important and G4-importance transcripts. (**a**) By normalising to the total length of different regions, 17.7%, 40.6%, and 41.7% G4s were found in the Region I (distal promoter, −2,000∼500 bp, black), II (proximal promoter, −500∼−1 bp, red) and III (downstream region, TSS∼1,000 bp, green) in TRRs of G4-less-important transcripts. (**b**) By normalising to the total length of different regions, 13.0%, 48.5%, and 38.5% G4s were found in the Region I (distal promoter, black), II (proximal promoter, red) and III (downstream region, green) in TRRs of G4-important transcripts. The proportion of G4s found in Region II, the proximal promoter region increases by 20% from G4-less-important to G4-important TRRs.

The correlation between *CF* and *CQ* scores (abundance and location significance of G4s in TRRs) were evaluated. As shown in Figure S3, *CF* and *CQ* are plotted as correlation maps. According to the maps, *CF* values are positively correlated to *CQ* values. Particularly, in G4-important TRRs (TRRs with both values higher than 50%), their G4 abundance is more positively correlated to their G4 location significance as compared to G4-less-important TRRs (TRRs with both values lower than 50%). To investigate whether this positive correlation of G4-important TRRs is only caused by the increase of G4 content or not, G4s found in different regions of TRRs were subjected to further investigation. The total number of G4s identified in different regions in G4-important and G4-less-important transcripts were counted and normalised to the length of different regions. If the increase of *CQ*, the location significance score, was solely dependent on the increase of *CF*, the G4 abundance score, then the proportion of the normalised total number of G4 found in different regions should be in constant between G4-important and G4-less-important TRRs. We found that the proportion of G4s found in the proximal promoter region (−500 bp to −1) was increased by 20% from less G4-important TRRs to G4-important TRRs (Figure 3). This indicates that the larger *CQ* value in G4-important TRR reflects the combined effects of increased total number of G4s and particularly the increased number of G4 in key promoter regions (with the high *W_k_* values). Most well-studied genes containing promoter G4s with potential regulatory functions are in the group of G4-important transcripts in the correlation map, including *MYC*, *VEGF-A*, *BCL-2*, *HIF1-α*, *MYB*, *PDGF-A*, *PDGFR-β*, *KRAS*, and *TERT* (Table S2). One interesting exception is *KIT*, with *CF*_o_ and *CQ*_o_ of 43.5% and 73.4%. This may suggest that G4 location might be more relevant than the abundance of G4 in evaluating potential biological significance of G4s in a TRR.

### G4-important Pathways

All pathways composed of more than 50 genes available in KEGG PATHWAY database were extracted, and *CF*_o_ and *CQ*_o_ values of each gene in a particular pathway were calculated. For genes with multiple transcripts, one transcript was picked randomly, and genes with both values (*CF*_o_ and *CQ*_o_) equaling to zero were excluded. Then, in each pathway, percentages of genes with TRRs having both *CF*_o_ and *CQ*_o_ values higher than 50% were counted. Pathways with more than 50% genes’ transcripts identified as G4-important were regarded as G4-important pathways as listed in Table 4. Ten iterations were carried out to evaluate effects from transcripts redundancy. For all pathways, no significant difference was found in *CF*_o_ and *CQ*_o_ values among iterations of each individual pathway (one-way ANOVA test, *P*≥0.05). Based on this criterion, G4-important pathways could be grouped into six categories with distinct functions. As shown in Table 4, the first category is cancer-related pathways where G4s are enriched in proto-oncogenes, in accordance with previously reported results [37]. Given the fact that G4s are enriched in cancer-related pathways, it is not difficult to understand why the off-target effects of certain G4-binding ligands with anti-cancer potential could well bring some benefits in anti-cancer therapy, such as the G4-binding fluoroquinolone derivative Quarfloxin [44], and the naphthalene diimide derivative 1 [40]. The second category is signaling pathways such as those involving VEGF, Wnt, Hedgehog, and MAPK (mitogen-activated protein kinase), which have key component containing promoter G4s with possible regulatory functions. Pathways related to cell junction, neuron function, and bacterial infection are also found to be G-important. The last category, heart function-related pathways, is found to be highly G4-important. There are seven pathways in the KEGG PATHWAY database involved in cardiac functions or heart diseases, while six of them are identified as highly G4-important. These pathways are not only involved in normal heart function such as calcium signaling and muscle contraction, but also in various cardiomyopathies, making targeting promoter G4s at pathway level rather than in single gene an attractive strategy (the multi-targeting strategy) in the treatment of certain cardiovascular diseases.

**Table 4:** KEGG pathways with high G4-importance.

KEGG Pathway ID	Pathway Description	% of High *CF*_o_and *CQ*_o_	No. of Gene	% of Gene with G4in TRR	*P*_max_ of *CQ*_o_ *	*P*_max_ of *CF*_o_ *
*Cancer-related pathways*						
hsa05210	Colorectal cancer	57.9±4.5	62	86.3±2.2	6E-4	0.001
hsa05211	Renal cell carcinoma	57.6±3.3	71	83.9±2.8	7E-4	0.003
hsa05213	Endometrial cancer	56.9±3.7	52	84.8±3.1	0.019	0.037
hsa05217	Basal cell carcinoma	53.7±2.9	55	93.8±1.3	4E-5	6E-6
hsa05200	Pathways in cancer	53.5±1.3	327	82.5±1.4	3E-11	3E-11
hsa05223	Non-small cell lung cancer	53.5±2.6	54	86.9±3.1	0.003	0.02
*Signaling pathways with promoter G4s in key components*						
hsa04340	Hedgehog signaling pathway	60.9±3.4	56	87.3±1.8	2E-4	1E-4
hsa04370	VEGF signaling pathway	60.7±3.0	80	87.9±2.1	8E-6	1E-4
hsa04010	MAPK signaling pathway	59.5±1.5	271	83.5±1.3	7E-16	1E-16
hsa04310	Wnt signaling pathway	58.9±2.5	150	89.9±0.9	2E-12	4E-12
*Cell junction-related pathways*						
hsa04520	Adherens junction	67.0±4.2	72	81.3±1.6	2E-5	6E-5
hsa04510	Focal adhesion	56.9±1.3	199	80.4±1.3	4E-8	8E-8
hsa04810	Regulation of actin cytoskeleton	55.7±2.4	213	79.1±2.3	3E-6	2E-5
hsa04530	Tight junction	53.9±2.1	129	77.0±1.8	8E-4	5E-4
hsa04540	Gap junction	53.8±2.0	88	86.1±1.9	8E-5	6E-5
*Neural cell regulation-related pathways*						
hsa04724	Glutamatergic synapse	61.8±1.6	129	87.1±1.6	5E-9	7E-9
hsa04360	Axon guidance	61.0±2.7	128	86.7±2.2	4E-9	4E-9
hsa04720	Long-term potentiation	60.2±2.0	70	90.6±1.2	1E-6	8E-6
hsa04722	Neurotrophin signaling pathway	55.6±2.1	125	88.9±1.9	4E-8	1E-7
hsa04912	GnRH signaling pathway	55.5±1.3	104	85.9±1.7	1E-6	1E-6
*Bacterial infection-related pathways*						
hsa05130	Pathogenic E.coli infection	58.6±4.9	55	82.7±3.8	0.004	0.008
hsa05131	Shigellosis	55.8±3.4	62	86.3±2.3	8E-5	2E-4
hsa05100	Bacterial invasion of epithelial cells	54.5±2.8	71	82.4±3.7	0.006	0.01
*Heart function-related pathways*						
hsa05412	Arrhythmogenic right ventricular cardiomyopathy	60.0±3.5	73	80.1±3.5	0.004	0.01
hsa05410	Hypertrophic cardiomyopathy	57.5±3.1	83	77.1±2.7	0.01	0.04
hsa05414	Dilated cardiomyopathy	57.2±3.1	90	78.2±2.8	0.009	0.02
hsa04020	Calcium signaling pathway	54.4±2.1	178	83.1±1.5	5E-6	5E-6
hsa04260	Cardiac muscle contraction	54.2±3.0	72	73.2±2.9	0.1	0.4
hsa04270	Vascular smooth muscle contraction	53.8±1.0	121	85.2±1.8	1E-7	9E-7

* *P*_max_ values are calculated by comparing the *CQ*_o_ and *CF*_o_ scores of genes from individual pathways with those from all transcripts without redundancy in each iteration by Wilcoxon rank sum test. *P*≤0.05 means the G4 abundance (*CF*_o_) or location significance (*CQ*_o_) of gene involved in the pathway is significantly different from those at genome level. For all pathways, no significant difference was found in *CF*_o_ and *CQ*_o_ values among iterations of each individual pathway (one-way ANOVA test, *P*≥0.05).

Based on the high importance of G4s in cardiac function related pathways, we assume that G4s might function as a general regulatory element in cardiac related genes. To validate our bioinformatics results and our assumption, we have chosen human cardiac troponin I (*TnIc*) as a model system to investigate possible biological functions of cardiac promoter G4s. As one of the central components in cardiac muscle contraction pathway, TnIc is also involved in most heart function related pathways. Furthermore, TRR of *cTnI* is highly G4 important according to our bioinformatics analysis (*CF*_o_ 92.6%, and *CQ*_o_ 95.2%). In the promoter of human *TnIc*, two types of G4-forming motifs were identified, which are designated as “MNSG4” (between −528 and −319 bp upstream to the TSS), and “−80 G4” (centred at −80 bp to the TSS) (Figure 1 a). Both MNSG4 and −80 G4 are characterised in solution and in HEK293 cells and the results are shown below.

### Evidence of G4s Formation in Solution

EMSA experiments were first used to probe the formation of G4s in the unlabeled MNS and −80 G4-forming sequences from the promoter of human *TnIc*. The fast migrating band of MNSG4-forming sequences (oligos **Tr_MNS_-I**) in native gels indicated that intramolecular G4(s) is the only conformation at 5 µM oligo concentration. However, at similar concentration, slow migrating bands of −80 G4-forming sequence (oligo **Tr_-80_-II**) were observed in a native gel, indicating it can adopt a mixture of intramolecular (the major form,∼60% according to band intensity) and intermolecular G4s. EMSA results are shown in Figure S4.

**Figure 4:**
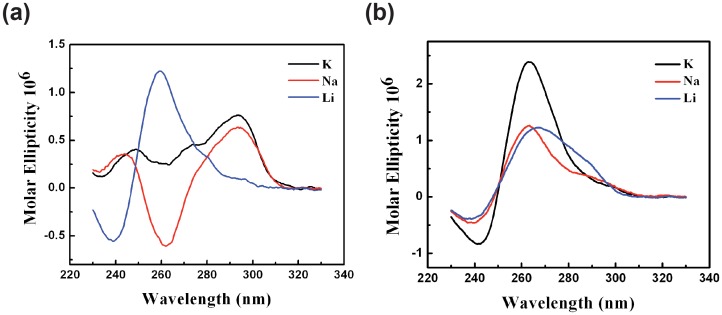
CD spectra of the two promoter G-quadruplex sequences in solutions containing different salts. (**a**) single-repeat MNSG4. (**b**) −80 G4.

Comparative CD spectra analysis was also used to provide primary evidence of the formation and general conformations of both MNSG4 and −80 G4 in solution. Briefly, a single repeat of MNSG4 (oligo **Tr_MNS_-I**) may adopt parallel structure(s) in LiCl solution. In KCl solution, it is very likely that the majority of single repeat of MNSG4 adopts a similar folding pattern as the TBA (thrombin binding aptamer) and forms an anti-parallel G4 (or G4s) [64], [65] (Figure 4 a). For the −80 G4, the −80 G4-forming sequence (oligo **Tr_-80_-I**) may adopt a hybrid parallel/anti-parallel structure similar to the BCL-2 Pu39WT in LiCl solution [30], while in NaCl and KCl solutions, it adopts typical parallel conformation (Figure 4 b). However, as an empirical method, CD spectra are not conclusive to confirm the existence of G4s in solution [66]. Indeed, CD spectra with a peak at 260 nm and a trough at 240 nm, which is believed to represent the existence of parallel G4s, can also be observed in duplexes, hairpins and single-stranded DNA samples [67]. Thus, to further confirm the existence of both MNSG4 and −80 G4, fast migrating bands from EMSA experiments (in the presence of 100 mM K^+^) were extracted and subjected to DMS footprinting.

According to the different protection effects in DMS footprinting, a preferred folding pattern of MNSG4 is proposed as G_3_N_5_G_3_N_5_G_3_N_2_G_3_, while for the −80 G4 multiple folding patterns may coexist with first loop ranging between 7∼10 nucleotides, and second and third loops ranging between 1∼2 nucleotides (Figure S5). Both of them are distinct from the general folding pattern of promoter G4s indentified in cancer-related genes. Although the exact G4 structures of MNSG4 and −80 G4 cannot be proposed based on DMS footprinting results alone, those fully protected guanines in both sequences clearly indicate the formation of G4s.

### Thermal Denaturation of MNSG4 and −80 G4 Measured by CD Melting

CD melting experiments were used to investigate the thermal stability of these G4s in the presence of 100 mM K^+^. For the MNSG4 (oligo **Tr_MNS_-I**), by fitting the melting curve obtained at 295 nm, the melting temperature and unfolding enthalpy and entropy of the anti-parallel MNSG4 were derived to be 71.4±0.5°C, 170±11 kJ mol^−1^ and 494±31 J mol^−1^ K^−1^ respectively by a model as previously reported (Figure S6 a). For the −80 G4 (oligo **Tr_-80_-I**), the parallel G4 with lower thermal stability, which accounts for 57.3% of CD signals at 263 nm, was almost fully denatured at 90°C, while no denaturing was observed for the other parallel species with high thermal stability. Since it has been shown by EMSA experiments that about 40% of the −80 G4 is able to adopt an intermolecular G4 conformation at 5 µM concentration, it is likely that the undenatured parallel species corresponds to intermolecular parallel G4s (Figure S4 b and S6 b).

### Unfolding Kinetics of MNSG4 and −80 G4 Measured by FRET

**Figure 5:**
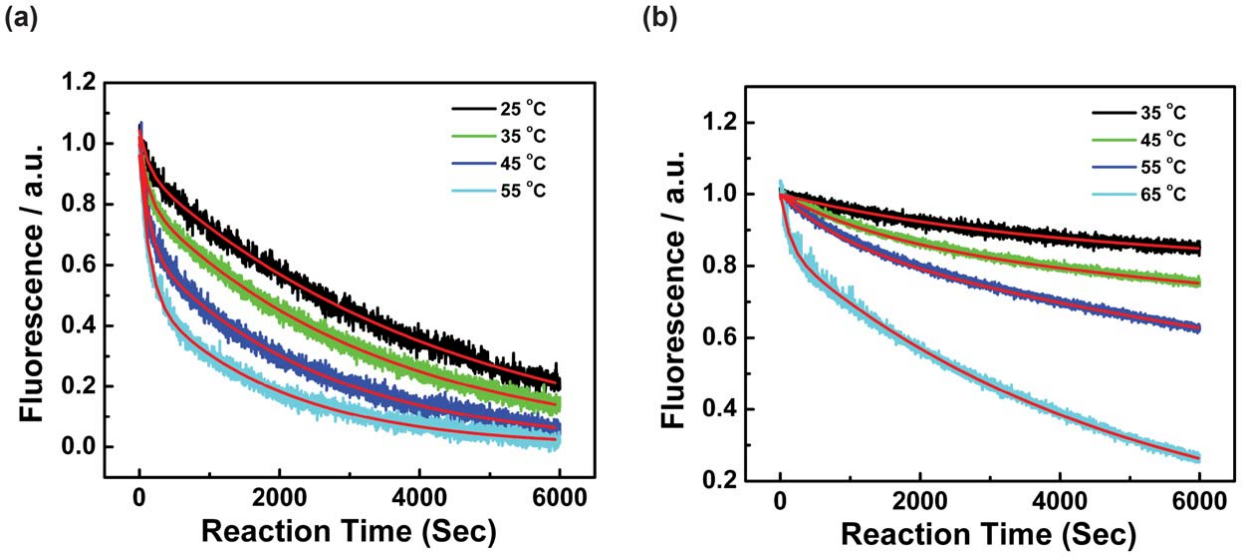
TnIc G4s unfolding curves and corresponding fitted curves. (**a**) Decrease of Cy5 fluorescence in the process of MNSG4 (formed between oligos **Tr_MNS_-III** and **Comp-Cy5**) opened by its C-rich complimentary strand (oligo **Tr_MNS_-II**) at different temperature. Black, green, blue and cyan curves are from experimental data obtained at 25, 35, 45, and 55°C, respectively, and red curves are fitted results by a double exponential decay model. (**b**) Decrease of Cy5 fluorescence in the process of −80 G4 (formed between oligos **Tr_-80_-III** and **Comp-Cy5**) opened by its C-rich complimentary strand (oligo **Tr_-80_-II**) at different temperature. Black, green, blue and cyan curves are from experimental data obtained at 35, 45, 55, and 65°C, respectively, and red curves are fitted results by a double exponential decay model. Hybridisation was performed between 100 nM G4 forming oligos and 2 µM complimentary C-rich oligo. The reaction was carried out in 100 mM KCl solution containing 10 mM Tris-HCl (pH 7.4).

Two subpopulations with different unfolding behaviors were revealed by fitting the unfolding curves of MNSG4, (Figure 5 a and Table S3). The fast hybridised subpopulation is believed to be partially folded or randomly coiled oligos with hybridisation rate constant around 0.4×10^4^ M^−1^s^−1^. As compared to a typical hybridisation rate constant, which is around 10^5^ M^−1^s^−1^, the much slower hybridisation rate constants observed here clearly indicated that the C-rich oligos still need to unfold certain unstable structures before fully hybridising the partially unfolding G-rich strand. The slow decaying component represents the unfolding process of MNSG4. As compared to the KIT1 G4 studies that used a similar system [68], the MNSG4 is more kinetically stable at high temperature (time constants of KIT1 G4 at 45 and 50°C are 2,300 and 1,000 s respectively), which echoes the higher thermal stability of MNSG4 in single-stranded form. The activation energy of the unfolding of MNSG4 was determined as 22.1±0.4 kJ mol^−1^ by Arrhenius equation (Figure S7 a and Table S3), and the activation enthalpy and entropy were derived by Eyring equation as 19.6±0.4 kJ mol^−1^, and −249.2±1.2 J mol^−1^ respectively. This relatively low activation energy/enthalpy and very negative entropy indicates that the unfolding of MNSG4 is entropically driven, and this may suggest that the MNSG4 unfolds through a “pre-organized” transition state, which possibly relates to the long loops in the folded MNSG4 (two 5-nucleotide adjacent loops). In the process of MNSG4 opening, these fluctuating long loops may partially anneal to the C-rich strand, resulting in a series of relatively “ordered” intermediates with negative entropy, and the invasion of C-rich strand may further destabilise the folded G4 structures without transiting G4 into other partially unfolded structures (more disordered structures with positive entropies).

Similar experiments were carried out on the TnIc −80 G4, and two subpopulations (fast and slow unfolding components) were revealed with different kinetic behaviours (Figure 5 b, Table S3). The fast-unfolding −80 G4 (−80 G4-F) exhibits comparable kinetic stability to the MNSG4, while the slow-unfolding −80 G4 (−80 G4-S) is at least 30-fold more stable than the −80 G4-F, and of similar stability as the KIT2 G4 studied previously by using an identical method [68]. Both −80 G4-F and −80 G4-S were characterised by a large activation enthalpy and small unfavoured activation entropy, suggesting the existence of more significant enthalpic barriers (Figure S7 b and Table S3). As compared to the MNSG4, only one long loop was found in the −80 G4 (the first loop, length between 10∼7 nucleotides), and correspondingly less negative activation entropies were identified in unfolding processes of the −80 G4. As the −80 G4-F has a more negative Δ*S* ^‡^ than the −80 G4-S, the destabilising contribution from the loop (partially annealed more ordered states) of −80 G4-F is more prominent than that from −80 G4-S, and thus less energy is required for the −80 G4-F to reach the transition state for the full hybridisation to happen. Indeed, a smaller Δ*H* ^‡^ of −80 G4-F was also found.

### Stabilisation of MNSG4 and −80 G4 by Synthetic G4-binding Ligands

**Figure 6:**
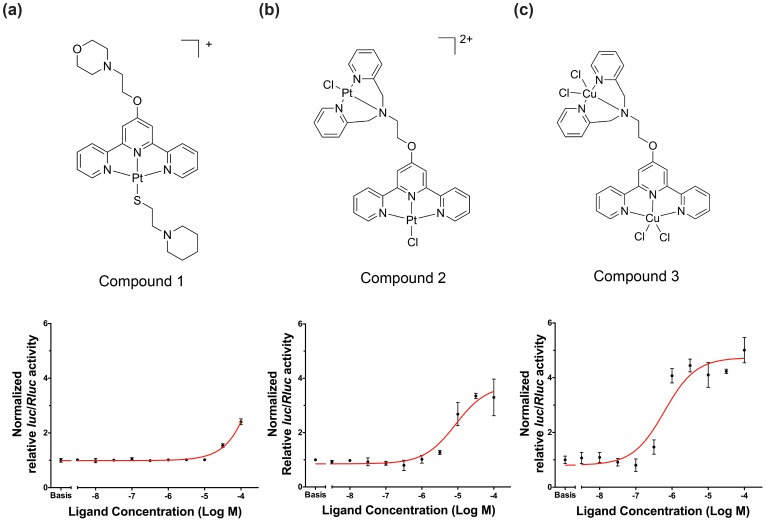
Structures and dose-response curves of compound 1, 2, and 3. (**a**) Compound **1** only slightly activated downstream luciferase expression (EC_50_ = 812.1 µM). (**b**) Compounds **2** moderately activated downstream luciferase expression (EC_50_ = 1.6 µM). (**c**) Compound **3** significantly activated downstream gene expression (EC_50_ = 0.6 µM). Experiments were carried out by measuring the firefly (hTnIc-6MNS(E)-WT) and *Renilla* luciferase ratio changes in the presence of ligand with different concentrations.

Once it was established that these sequences can indeed form quadruplex structures, it was of interest to determine whether small molecules could facilitate the formation and stabilise the quadruplex structure. Over the past few years some of us have demonstrated that metal-containing compounds (where the metal is tightly bound to an organic ligand) can be excellent quadruplex DNA binder [55], [69]. Therefore, we evaluated the ability of three of these metal compounds (compound **1**–**3**, Figure 6) to stabilise the quadruplex structures in the MNSG4 and −80 G4 sequences. The compounds were selected since they were found to be stronger quadruplex binders via a combination of Π–Π end-stacking interactions (with the terminal guanine tetrad) and electrostatic interactions (with the loops and grooves of quadruplexes) [55].

We first determined their relative binding affinity towards both quadruplexes via the well-established Fluorescent Intercalator Displacement (FID) assay. The results, which are summarized in Table 5, show that the two di-metallic compounds (**2** and **3**) bind strongly to MNSG4 with DC50 values under the 0.5 µM threshold for good binders. The mono-platinum compound **1**, also displays some interaction but its DC50 value is twice as big as those of **2** and **3**. Interestingly, there is a wider range of binding affinities towards the −80 G4 quadruplex with the di-copper compound (**3**) displaying a very low DC50 (i.e. strong binding), reasonably good affinity of **1** and poor affinity of di-platinum compound **2**. Furthermore, we had reported that these compounds are very selective binders to G4 DNA over duplex DNA (up to 100-fold selectivity [55]).

**Table 5:** ^MSN1^DC_50_ and ^−80 G4^DC_50_ values (µM) determined using FID assay for complexes 1–3 (values are average of three independent measurements and error is estimated to be below 5%).

Compound	^MSN1^DC_50_		^−80 G4^DC_50_		
1		0.76		0.55	
2		0.39		2.22	
3		0.31		0.19	

Thus, given the good binding affinity and selectivity of the di-copper compound to G4s, its G4-stabilising ability was further investigated by the CD melting experiment (by measuring the changes of G4 melting temperature, Δ*T*_m_) in the presence of the ligand. In 10 µM compound **3**, the CD spectrum of MNSG4 dramatically changed from parallel to hybrid of parallel/anti-parallel. The melting temperatures measured at 263 nm and 295 nm are 90.4±0.7°C and 86.2±2.1°C, respectively (Figure S8 a and b). It is likely that the di-copper compound preferentially binds to parallel MNSG4 structures (possibly due to the exposed end G-tetrads in parallel conformations), and thus promotes the transition of MNSG4 from anti-parallel to parallel structures. For the −80 G4, the presence of the compound did not change its CD spectrum. Due to the strong stabilising ability of the compound, only a small fraction of the parallel −80 G4 was melted at 93°C, and the melting temperature is estimated to be over 100°C (Figure S8 c and d). The binding preference to parallel G4s observed here might be due to the long loops in both MNSG4 and −80 G4. Long lateral or diagonal loops may partially or fully cover the end G-tetrad, and impede the Π–Π stacking, while the long external loops could leave enough space for the π–π stacking at the end G-tetrad for the first metal moiety, and even provide ideal electrostatic interactions sites for the second metal moiety.

### Ligand Screening in Cell Model

Since these ligands were able to bind to the MNSG4 and −80 G4 in solution with relatively high affinity and selectivity, we aim to investigate possible biological functions of these G4-binding ligands and their interactions with these G4 elements in living cells. Since both MNSG4 and −80 G4 are only conserved in *TnIc* promoters in the primate genomes, HEK293 cell line, rather than rat/mouse cardiomyocytes, was used. Dose-response curves of compound **1**–**3** were generated by measuring the firefly (vector hTnIc-6MNS(E)-WT) and *Renilla* luciferase ratios of lysates of transfected HEK293 cells incubated in the presence of ligands at different concentrations. Vector hTnIc-6MNS(E)-WT was used because it contains the greater number of G4 repeats, which was predicted to give the most significant response if the ligand-stabilisation of G4s is involved in determining the promoter activity. As shown in Figure 6 a, the mono-platinum compound **1**, showed a weak activating effect towards downstream gene transcription with the promoter activity being increased 2-fold at 100 µM concentration (EC_50_ = 812 µM). The di-platinum compound **2** exhibited modest activating effects towards downstream luciferase transcription with the promoter activity being increased by more than 3 times at 100 µM concentration (EC_50_ = 8.6 µM) (Figure 6 b). By replacing platinum with copper, the third compound (compound **3**) demonstrated most significant activation effect, which elevated the promoter activity by approximately 5-fold at 100 µM concentration (EC_50_ = 0.6 µM) (Figure 6 c). These results are in good agreement with the FID results that compound **3** is the best G4-binder among the three. Together, these observations suggest that the di-copper compound **3** has the best performance in living cells.

### G4-binding Ligand is Critical in Promoting Positive Regulatory Functions of *TnIc* Promoter G4s

**Figure 7:**
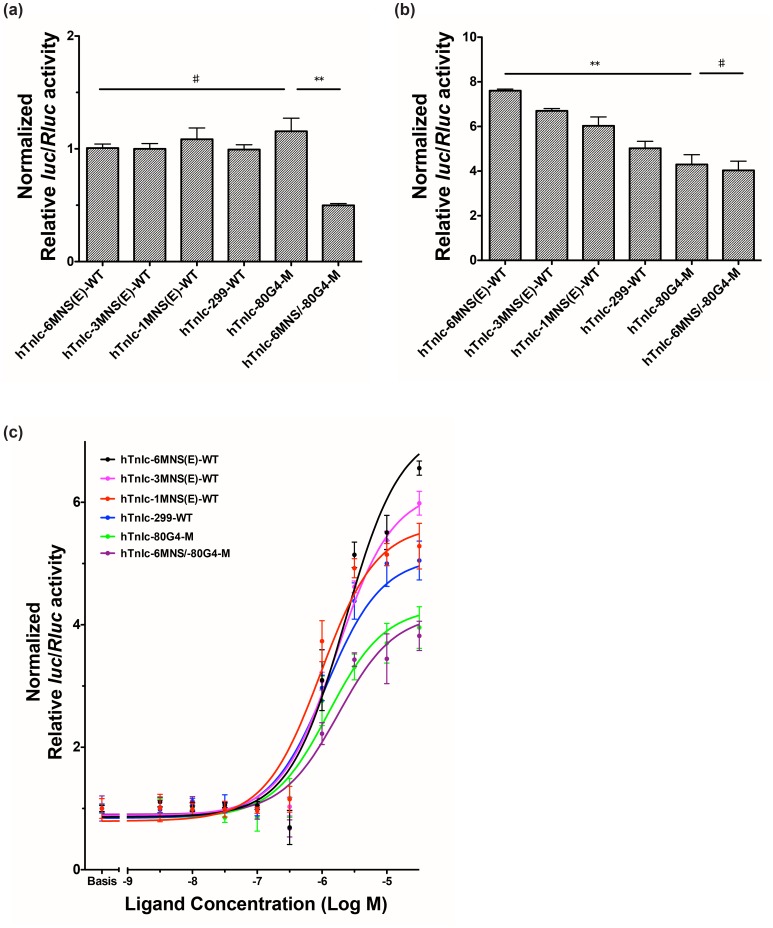
Transcription activity changes of human *TnIc* promoters in the presence of compound 3. (**a**) Transcription activity of constructed human *TnIc* promoters in the absence of compound **3**. (**b**) Transcription activity of constructed human *TnIc* promoters in the presence of 100 µM compound **3**. Columns in a and b are normalised to the luciferase transcription level of the wild type control 6MNS(E)-WT in the absence of ligand. (**c**) Dose-response curves of compound **3** with different luciferase vectors. (**P<0.01, *P<0.05 and ♯*P*≥0.05, no significant differences compared to WT construct).

To investigate the regulatory function of *TnIc* promoter G4s, firefly and *Renilla* luciferase ratios of different vectors were measured following incubation with and without the G4-binding ligand compound **3**. We found that compared to the engineered wild type construct containing six repeats of consensus MNSG4, human *TnIc* constructs containing three, one repeat, or no repeat, exhibited similar activities in driving downstream luciferase transcription (*P*≥0.05, Figure 7 a and Figure S9). This indicates that in the absence of G4-binding ligands, neither the MNSG4 nor Sp1 binding sites in the MNSG4 element appear to be involved in regulating downstream gene transcription, at least in HEK293 cells.

Regulatory roles of the −80 G4 together with a conserved Sp1 binding site in the promoter of human *TnIc* were also investigated by dual luciferase reporter assays in the HEK293 cell line in the absence of G4-binding ligand. As shown in Figure 7 a and Figure S10, the human *TnIc* construct containing −80 G4 with disrupted G4-forming sequence but intact Sp1 binding site (hTnIc-80 G4-M) has similar transcription activity to the engineered wild type promoter (hTnIc-6MNS(E)-WT). However, when the Sp1 binding site is mutated (hTnIc-80Sp1-M), transcriptional activity decreases by∼25%. Furthermore, when both the −80 G4-forming sequence and Sp1 binding site are mutated (hTnIc-80 G4/Sp1-M, Figure S10), transcriptional activity further decreases to 50% compared to the engineered wild type promoter. This may relate to the further mutated Sp1 binding site in vector hTnIc-80 G4/Sp1-M as compared to that in vectors hTnIc-80Sp1-M, rather than the disruption of the −80 G4 formation. Although the −80 G4 is not directly involved in regulating downstream gene transcription, the Sp1 binding site seems to be critical in determining downstream gene expression. This is similar to the situation where an Sp1 binding site found in the G4-forming sequence (KIT1) in the promoter of human *KIT* gene appears to be critical in determining the maximal activity of the human *KIT* promoter [70].

The −80 G4 mutation (with an intact Sp1 binding site, hTnIc-80 G4-M) which disrupts G4 formation, slightly elevated the transcriptional activity of *TnIc* promoter. The overall G4 mutant, hTnIc-6MNS/−80 G4-M, exhibited lowest transcriptional activity (Figure 7 a). The decreased luciferase expression from this vector may result from a combination of multiple mutation sites in MNSG4 and −80 G4, which have disrupted the critical Sp1 binding site within the −80 G4, and may have introduced certain inhibitory factor binding sites.

We conclude that in the absence of G4-binding ligand, both the MNSG4 and −80 G4 are not involved in regulating downstream gene transcription in HEK293 cells, and the depressed transcription levels mainly relate to the disruption of the critical Sp1 binding site within −80 G4. As *TnIc* is exclusively expressed in cardiomyocytes, we suspect that the formation and stabilisation of these promoter G4s may be associated with certain cardiac-specific proteins or TFs, which are absent in HEK293 cells. Since it has been reported that short proximal promoter of human *TnIc* is sufficient to confer cardiac-specific expression [71], it is possible that the luciferase expression level of these vectors without ligands is just the basal expression level in HEK293 cells. To test this hypothesis, luciferase assays were repeated in the presence of 100 µM compound **3**. As shown in Figure 7 b, when incubated with compound **3** for 24 hours before cell lysis, the expression activities of luciferase from all vectors increase differently. Generally, the transcription activity of human *TnIc* promoter gradually decreases when the number of MNSG4 repeat decreases, while the lowest transcription activities were observed in vectors with mutated −80 G4. It’s worth noting that in the presence of ligand, the overall mutant, hTnIc-6MNS/−80 G4-M, was also activated for about 4 times as compared to the wild type construct hTnIc-6MNS(E)-WT in the absence of ligand. This may relate to the interaction between the ligand and a G-rich region in the *TnIc* promoter (between −260 and −150 bp), which may adopt multiple two-tetrad G4s.

To further discriminate activation effects of MNSG4 and −80 G4, dose-response curves of different luciferase vectors treated with compound **3** were generated. As shown in Figure 7 c, the strongest activation effect was observed in the vector with wild type MNSG4 and −80 G4 (hTnIc-6MNS(E)-WT), in which downstream luciferase expression increases 6.5 times in the presence of 3.2 µM compound **3**. When the −80 G4 is intact, the activation effects of downstream luciferase decreases proportionally with the decrease of repeat number of MNSG4 (hTnIc-6MNS(E)-WT>hTnIc-3MNS(E)-WT>hTnIc-1MNS(E)-WT>hTnIc-299-WT). However, when the −80 G4 is mutated (hTnIc-80 G4-M, hTnIc-6MNS/−80 G4-M), lowest activation effects were observed again. These results suggest that the −80 G4, which is located in a region with high location significance (according to our hypothesis in bioinformatics studies), may operate as a gatekeeper in regulating downstream luciferase expression in HEK293 cells; the MNSG4, on the other hand, acts as to a fine tune regulation when the −80 G4 is intact. It is likely that both types of G4s orchestrate the gene expression of cardiac troponin I in the heart.

## Discussion

The correlations between G4s and gene functions, and the regulatory roles of promoter G4s in proto-oncogenes have been thoroughly investigated previously, which has led to the application of several G4-binding ligands as potential anticancer drugs (as reviewed in [38], [72]). There is now compelling evidence to support the existence of G4 *in vivo*, such as G4-induced guanine-protection revealed by *in vivo* DMS footprinting [23] (reviewed in [73]). However, although it is clear that G4 complexes function in living cells and there has been some good successes in designing specific G4 binding small molecule ligands (reviewed in [38]), discriminating and targeting specific G4s via small synthetic G4-binding ligands remains very challenging. Such efforts are hampered by many factors including: (i) structural polymorphism and dynamic properties of G4s, (ii) similar major recognition sites (G-tetrad) in different G4s, (iii) limited precise structural data of G4s available, (iv) low abundance and instability of G4s in the human genome as compared to duplex DNA, (v) obstacles in synthesis of natural G4-binding ligands and (vi) poor pharmaceutical properties of some synthetic compounds. Among all these issues, the selectivity of G4-binding ligands is the most notable one that warrants further improvement. Although there are some ligands reported to interact with G4s much more selectively than with duplex DNA, such as Mn^III^ porphyrin (10^4^-fold) [74], and dimetallic terpyridine-based ligand (over 100-fold) [55], the selectivity of most ligands are in the range between 10- and 100-fold (reviewed in [38], [39]). Furthermore, very few ligands have been reported to exert satisfactory selectivity between different G4s. Thus, we reason that G4-binding ligands with ideal selectivity and affinity to a particular promoter G4 structure would be difficult to achieve at the moment, and off-target effects would be inevitable, limiting the therapeutic potential of these ligands *in vivo*. However, off-target effects or multi-targeting effects of G4-binding ligands may in turn become advantageous and enhance their therapeutic potential in some cases as mentioned in the Introduction section. This implies that targeting multiple similar G4s in a particular pathway might be feasible, and furthermore, this could be an alternative to overcome selectivity problem of G4 ligands. Therefore, G4-pathway correlation is particularly important in applying G4s as potential targets for the treatment of certain diseases.

In this study, we first investigated the abundance of G4s in the TRRs of genes active in different tissues. The tissue-specific and location-specific enrichment of G4s in TRRs of genes were clearly shown, particularly for the genes active in the heart. Enrichment of G4s in different regions around TSS is known to associate with diverse biological processes. For example, G4s in the distal or proximal promoters of proto-oncogenes normally act as repressors in regulating downstream gene transcription (*MYC* promoter G4 [10], [11]). G4s in the downstream regions relative to TSS can modulate mRNA translation efficiency differently depending on the strand polarity [35], [36]. Thus, this tissue-specific, location-specific, and asymmetric G4 enrichment may also imply that tissues with high G4 abundance in TRRs or certain regions of TRRs have evolved certain tissue-specific mechanisms to tolerate or even to utilize G4s in the processes of transcription regulation.

Previous genome-wide studies have found that promoter G4s have a strong positional bias towards TSS in different species [6], [35], [59], [61], [75], [76]. This positional bias of promoter G4 is believed to be a result from evolution selection [6], [37]. Thus, in addition to G4 abundance (*F* score, and *CF*), the location significance of G4 (*Q* score, and *CQ*) is also considered when evaluating the biological importance of G4s in each TRR. Generally, correlation between *F* and *Q* scores in G4-less-important TRRs is not as strong as that in G4-important TRRs. Furthermore, we notice that when G4 importance increases, the probability of finding G4s in the proximal (−500 bp∼−1 bp) promoter regions of TRR increases. Because the majority of promoter G4s with proven regulatory functions locate in the proximal promoter regions, it is highly likely that the accumulation of proximal promoter G4s in pathways with high G4 importance is biologically relevant. In regions downstream the TSS, however, the probability is relatively constant, suggesting that the transcription induced G4 formation might be ubiquitous in different genes.

G4-important pathways show significant connection with their functions (Table 4). Particularly, in accordance to previous findings, most cancer-related pathways are also G4-important, which may suggest that off-target effects of certain anticancer G4-binding ligands can provide extra benefits for cancer treatment. Heart function-related pathways, especially those involved in various cardiomyopathies, are also found to be highly G4-important, indicating the therapeutic potential of G4s in cardiovascular diseases.

Because of its high G4 importance and unique expression pattern, the proximal promoter of human *TnIc* was chosen as a model to investigate possible regulatory functions of G4s in cardiac function-related genes. Two G4 elements, the MNSG4 and −80 G4 were analysed separately. Results from CD, EMSA and DMS footprinting experiments have confirmed formation of these G4 elements in solution. Different folding patterns between these cardiac promoter G4s and proto-oncogene promoter G4s may relate to their distinct biological functions and therefore warrant further investigation by NMR spectroscopy.

The stability of MNSG4 and −80 G4 were both evaluated by their unfolding processes either thermodynamically and kinetically. Both have similar thermal stability (Table 6) as compared to the human telomeric G4 [77], [78], and other G4s with similar loop length, such as MYC, KIT, WNT1 and VEGF G4s [4]. Kinetic stability studies (Table S3) revealed that the unfolding of MNSG4 is entropically driven and that it can unfold relatively easily [79], [80]. On the other hand, the unfolding of the −80 G4s is enthalpy-driven. According to limited kinetic stability data of other G4s, the MNSG4 and the fast unfolding −80 G4 are slightly less kinetically stable than the others, while the slow unfolding −80 G4 is much more stable than the others (*Oxytricha* telomeric G4 [81], KIT1 G4 [68], KIT2 G4 [68], and human telomeric G4 [60]). As the biological roles of human telomeric, *MYC*, *VEGF*, and *KIT* G4s have already been established, it is reasonable to assume that both TnIc MNSG4 and −80 G4 are stable enough to possess certain biological roles in the promoter of human *TnIc* in the context of adjacent duplex.

**Table 6:** Energetics of TnIc MNSG4 and −80 G4 unfolding in solutions determined by CD melting.

Oligo Names	Wavelength/nm	*T*_m_/°C	Δ*H*/kJ mol^−1^	Δ*S*/J mol^−1^ K^−1^	Δ*G*_(310 K)_/kJ mol^−1^
Tr_MNS_-I (TnIc MNSG4)	263	N/A	N/A	N/A	N/A
	295	71.4±0.5	170±11	494±31	17.2±1.3
Tr_-80_-I (TnIc −80 G4)	263	75.2±1.0	187±22	538±62	20.5±1.7
	295	N/A	N/A	N/A	N/A

Experiments were carried out in solutions containing 10 mM Tris-HCl (pH 7.4) and 100 mM K^+^.

Dose response curves from different constructs demonstrated that the stabilisation of −80 G4 is critical in activating downstream luciferase expression, while the individual MNSG4 acts as a fine-tuner. Since in HEK293 cells, most cardiac specific proteins and transcription factors are absent, this activation effect may be due to the negative twist from the formation of G4s in the promoter region, which can facilitate the unwinding of supercoiled plasmid DNA. The regulatory roles of G4 elements in human *TnIc* promoter were mainly measured by comparing the transcription activities of engineered human *TnIc* promoter in HEK293 cells with and without the G4-binding ligand by dual luciferase reporter assays. This is based on the assumption that the G4-binding ligand, compound **3**, is able to directly interact with these TnIc G4 elements in living cells, and subsequently to induce transcription activity changes. However, similar to most studies published so far, direct evidence indicating the interaction between G4-binding ligands and G4s in living cells is extremely difficult to obtain and could not be obtained in this study. However, several pieces of indirect evidence support the conclusion that the positive regulatory roles of these *TnIc* promoter G4s relate to the stabilisation of these elements by the ligand. The first evidence is the selectivity of the ligand to the TnIc G4 elements as compared to the dsDNA. Previous studies showed that compound **3** exhibits a relatively high selectivity to G4s as compared to dsDNA (∼ 100-fold [55]), although its selectivity among different G4s is relatively low. These features of compound **3** eliminate the doubts that this ligand may interact with dsDNA, and change the transcription activity of *TnIc* promoter in HEK293 cells indirectly (such as the inhibitory effects of Quarfloxin towards *MYC* [82], [83]). The second evidence is the preference of the ligand between the TnIc MNSG4 and −80 G4. Biophysical studies of G4-ligand interactions indicated that the Cu-Cu compound has better performance when interacting with the −80 G4. Similarly, in cell studies, activation effects from the ligand-stabilised −80 G4 are more prominent. Finally, the prevalent activating effect from −80 G4 in regulating downstream gene expression is in good agreement with our hypothesis in the bioinformatics studies about location significance of promoter G4s (G4s found in the region with high distribution probability are more important). Thus, although additional experiments may be necessary to fully clarify the interaction between compound 3 and TnIc G4 elements in the context of a living cell, it is very likely that the activation effects of these G4 elements are directly induced by interacting with the G4-binding ligand.

In contrast to the widely observed G4-mediated phenomena in promoters of oncogenes that formation and stabilisation of these promoter G4s could impede downstream gene transcription, we found that the formation and stabilisation of the MNSG4 and −80 G4 could activate downstream gene transcription. This is similar to the G4s found in the ILPR [8] and skeletal muscle gene promoters [84]. The formation and functions of G4s around gene promoters are dependent on local environment (transcription-induced supercoiling, existence of transcription bubbles, etc.), and the availability of G4-interacting proteins in a particular cell type. Thus, we believe that the actual function of individual promoter G4 needs to be studied case by case. Here we suggest two possible mechanisms for this G4-mediated transactivation.

The first mechanism is related to the superhelicity of the testing plasmid. It has been known that the formation of G4 structures within the DNase I or S1 nuclease hypersensitive pPu/pPy tracts in gene promoters are often associated with negative supercoiling stress, which facilitates the local unwinding or melting of duplex DNA [21], [85], [86], such as in cases of ILPR [87]–[89], VEGF [23], [24] and MYC [90]. Furthermore, the RNA polymerase (RNApol) can generate negative supercoiling during transcription [91]. It is worth noting that this transcription-induced supercoiling force was found to enable DNA transitions to conformations other than B-DNA in an *in vivo* study of the MYC promoter [92]. Therefore, it is possible that in the case of human *TnIc* promoter, the stabilisation of multiple G4s by the ligand facilitates the local unwinding of the negatively supercoiled plasmid, and resolves the transcription-induced supercoiling forces, hence increasing transcription rate. Since the −80 G4-forming motif locates very close to the TSS, the formation of this G4 may exert stronger forces to release the transcription-induced supercoiling stresses, as compared to the MNSG4. In addition, the formation of the −80 G4 may result in the exposure of multiple TF binding sites around it and facilitate the initiation of the transcription. This may explain the “gatekeeper” role of the −80 G4 and “fine-tuner” role of the MNSG4. We are aware that *in vitro* and *in vivo* DMS footprinting [23], [24] to probe the formation of these G4 elements would be necessary to fully characterise this mechanism.

The G4/E-box-mediated mechanism is another possibility. Recently, Shklover et al. proposed that the promoter G4s formed in muscle-specific genes may function as binding sites for homodimeric MyoD and increase its concentration in the micro-environment around the promoters [84]. This accumulated homodimeric MyoD then associate with constitutive E-proteins to form heterodimers, which bind to adjacent E-box (E47) and activate downstream gene expression [84]. Interestingly, in the promoter of human *TnIc*, potential E-boxes are also identified around both MNSG4 and −80 G4 [49]. In HEK293 cells, these co-localized G4-forming motifs and adjacent E-boxes may provide binding sites for constitutively expressed bHLH factors and E-proteins, and hence transactivate the downstream luciferase gene. This mechanism may also exist in the human cardiomyocyte, since several cardiac-restricted bHLH proteins have been found to be important in heart development, such as HAND1 and HAND2 [93]–[95].

In summary, this study shows that potential G4-forming motifs are highly enriched in genes active in the heart as well as those involved in cardiac function-related pathways. As shown in the model of *TnIc* promoter, it is very likely that multiple G4s act cooperatively in regulating downstream gene transcription. Thus, multi-targeting strategy via promoter G4s could be utilised in manipulating certain gene expression or even modulating pathway performance. On the other hand, it is necessary that all the G4 forming motifs in a given promoter are carefully investigated to understand their potential regulatory functions. It should be noted that HEK293 cell line used in this study is a limited cell model, but we have shown that it is still valuable to understand the ligand-induced promoter activity changes of human *TnIc*. Cardiomyocytes could be used in future studies to fully resolve the G4-mediated regulatory mechanism.

## Supporting Information

Figure S1 **Distribution of G4 abundance scores (the** ***F*** **scores) and corresponding cumulative frequencies (the** ***CF*** **scores) on coding strand (a), template strand (b), and both strands (c).** In the analysis, TRRs from 46,205 transcripts exported from ENSEMBL with redundancy were included. TRRs of transcripts without G4-forming motifs were excluded in calculating the distribution and corresponding cumulative frequency.
(DOC)

Figure S2 **Probability distribution and location significance scores (the** ***Q*** **scores) distribution of G4s in TRRs of transcripts.** (**a**) Frequency of PQS (potential quadruplex sequence) at each position (*W_k_* as described in Equation 3) on coding strand (black), template strand (red), and both strands (green) in TRRs of all transcripts. TRRs from 46,205 transcripts exported from ENSEMBL with redundancy were included. (**b∼d**) Distribution of G4 location significance (lower graphs) and corresponding cumulative frequency (upper graphs) of all transcripts available in ENSEMBL database on the coding strands, template strand, and both strands, respectively. TRRs of transcripts without G4-forming motifs were excluded in calculating the distribution and corresponding cumulative frequency.
(DOC)

Figure S3 **Correlation between the location significance (the** ***CQ*** **score) and abundance (the** ***CF*** **socre) of G4s identified in TRRs of all transcripts with redundancy.** (a∼c) Correlation between G4 location significance and G4 abundance on the coding strand (*CQ*_cd_ vs. *CF*_cd_), template strand (*CQ*_ncd_ vs. *CF*_ncd_), and both strands (*CQ*_o_ vs. *CF*_o_), respectively. In each map, transcripts with the TRRs *CQ* and *CF* scores larger than 50% are identified as G4-important transcripts, and those with both values smaller than 50% are G4-less-important (or less G4-important) transcripts. Correlation coefficients between *CF* and *CQ* on coding strand (*CF*_cd_ vs. *CQ*_cd_), template strand (*CF*_ncd_ vs. *CQ*_ncd_), and both strands (*CF*_o_ vs. *CQ*_o_) are 0.57, 0.50, and 0.61, respectively. However, corresponding correlation coefficients for G4-important TRRs are 0.61 (coding strand), 0.58 (template strand), and 0.66 (both strands), respectively, while those for G4-less important TRRs are 0.10, 0.09, and 0.34 respectively. Apparently, in G4-important TRRs, their G4 abundance is more positively correlated to their G4 location significance.
(DOC)

Figure S4 **Native and denaturing gel electrophoresis indicating the formation of G4s.** (**a**) TnIc MNSG4, 1 repeat: Lane1 and 2 are oligos **Tr_MNS_-II** (C-rich) and **Tr_MNS_-I** (G-rich) run in a native gel. Lane 3 and 4 are oligos run in a denaturing gel as controls. (**b**) TnIc −80 G4: Lane1 and 2 are oligos **Tr_-80_-II** (C-rich) and **Tr_-80_-I** (G-rich) run in a native gel. Lane 3 and 4 are oligos run in a denaturing gel as controls. Formation of intramolecular G4s is proved by species with fast mobility under native conditions (indicated by black arrows) but not under denaturing conditions. Intermolecular G4s formed by the −80 G4 forming sequence are indicated by gray arrows in (**b**).
(DOC)

Figure S5 **DMS footprinting results of TnIc MNSG4 and −80 G4.** (**a**) DMS footprinting of MNSG4 (oligo **Tr_MNS_-I**). Strong DMS protection is observed at G1∼G3, G5∼G7, G11∼G13, and G14∼G15. Two guanines at the 3′ of MNSG4 (G17∼18, oligo **Tr_MNS_-I**) are not involved in G4 formation. Compared to other guanines involved in loops (G4, G8, and G9), G10 is partially protected from DMS. Because G13 is fully protected from DMS, this partial protection of G10 may be due to the gyration hindrance from the adjacent G4 structure, which blocks its access by DMS. (**b**) DMS footprinting of −80 G4 (oligo **Tr_MNS_-I**). In the first 5′ run of guanines (G1∼G5), partial protection of guanines is apparent. Meanwhile, in the second and third runs of guanines (G9∼G12 and G13∼G16), partially protected flanking guanines in each G-run are also found (G9/G12 and G12/G16) with two fully protected guanines in the middle (G10∼G11 and G14∼G15). This poorly defined footprinting pattern of −80 G4 element is possibly due to the long first loop (at least 7 nucleotides) which induces flexibility of the G4 conformations. Together with CD experiments, it’s highly likely that this sequence can fold into a mixture of exchanging parallel intramolecular G4s with different loop arrangements in KCl solution. Furthermore, in the second and third G-runs, it is clear that G12 and G13 are better protected from DMS than G9 and G16, which may reflect the preference of different intramolecular G4s.
(DOC)

Figure S6 **CD melting of the two promoter G-quadruplex sequences. (a)** single-repeat MNSG4. **(b)** −80 G4.
(DOC)

Figure S7 **Arrhenius plot for unfolding of TnIc G4s.** (**a**) Arrhenius plot for the slow decaying component identified in the unfolding process of TnIc MNSG4. Empty circles represent opening rates measured at different temperatures, and red line is the result fitted by Arrhenius equation. The activation energy is determined to be 22.1±0.4 kJ mol^−1^. (**b**) Arrhenius plot for the slow and fast decaying components identified in the unfolding process of TnIc −80 G4. Empty triangles and squares represent opening rates of the fast and slow decaying components, respectively, and red lines are results fitted by Arrhenius equation. The activation energies for the fast and slow decaying components are 86.1±23.7 and 100.6±6.6 kJ mol^−1^, respectively, as determined by the fits.
(DOC)

Figure S8 **CD melting of TnIc MNSG4 and −80 G4 in the presence of the G4-binding ligand (complex 3).** (**a**) CD melting spectra of TnIc MNSG4 (oligo **Tr_MNS_-I**, 5 µM**)** in the presence of 10 µM complex **3**. (**b**) CD signal changes of TnIc MNSG4 (oligo **Tr_MNS_-I)** at 295 nm (black dots) and 263 (red dots) nm wavelengths in response to temperature changes, both of which were fitted by a sigmoidal model (indicated by red line and black line respectively). The melting temperature measured at 265 nm and 295 nm are 90.4±0.7°C and 86.2±2.1°C respectively (**c**) CD melting spectra of TnIc −80 G4 (oligo **Tr_-80_-I**, 5 µM**)** in the presence of 10 µM complex **3**. (**d**) CD signal changes of TnIc −80 G4 (oligo **Tr_-80_-I)** at 295 nm (black dots) and 263 (red dots) nm wavelengths in response to temperature changes, both of which were fitted by a sigmoidal model (indicated by red line and black line respectively). All experiments were carried out in 10 mM Tris-HCl buffer (pH 7.4) containing 100 mM K^+^.
(DOC)

Figure S9 **Comparison of the transcription activities of human** ***TnIc*** **promoters containing variable number of MNSG4.** Transcription activities of human *TnIc* promoters containing 6 repeats of consensus MNSG4 (hTnIc-6MNS(E)-WT), 3 repeats of consensus MNSG4 (hTnIc-3MNS(E)-WT), 1 repeat of consensus MNSG4 (hTnIc-1MNS(E)-WT), and no repeat of MNSG4 (hTnIc-299-WT). No transcription activity difference was found among these human *TnIc* promoters (*P*≥0.05, no significant differences).
(DOC)

Figure S10 **Transcription activities of human** ***TnIc*** **promoters with wild type and mutated −80 G4.** Mutation of −80 G4 alone (hTnIc-80 G4M) did not change the transcription activity, while the mutation of Sp1 binding site (hTnIc-80sp1M-1/2) led to∼25% depression in the transcription activity. When both G4 formation and Sp1 binding sites were mutated (hTnIc-80 G4/sp1M), 50% decrease of the transcription activity was found (* *P*<0.05 and ♯ *P*≥0.05 no significant differences).
(DOC)

Table S1 **The rankings and scores of G4 enrichment in TRRs of genes active in different tissues.** G4s in the full-length TRR (Whole TRR), distal promoter region (−2,000∼−501 bp), proximal promoter region (−500∼−1 bp), and downstream region (TSS∼+1,000 bp) were searched and the procedure was repeated randomly for five times.
(DOC)

Table S2 ***CF*** **_o_ and** ***CQ*** **_o_ values of TRRs of genes containing well-studied promoter G4s.**
(DOC)

Table S3 **Parameters obtained from curve fittings of TnIc MNSG4 and −80 G4 unfolding in solution.**
(DOC)

Protocols S1 **Supporting protocols including G4-binding ligand synthesis, Electrophoretic mobility shift assay, CD characterization, DMS footprinting, unfolding kinetics and the human genome sequence data bases used in the bioinformatic analysis.**
(DOC)
