# Supplementary material for: Possible Regulatory Roles of Promoter G-Quadruplexes in Cardiac Function-Related Genes – Human TnIc as a Model
Source: PLoS One. 2013 Jan 9;8(1):e53137. doi: 10.1371/journal.pone.0053137 (PMC3541360; doi:10.1371/journal.pone.0053137)
Supplement: Figure S1 — Distribution of G4 abundance scores (the F scores) and corresponding cumulative frequencies (the CF scores) on coding strand (a), template strand (b), and both strands (c). In the analysis, TRRs from 46,205 transcripts exported from ENSEMBL with redundancy were included. TRRs of transcripts without G4-forming motifs were excluded in calculating the distribution and corresponding cumulative frequency. (DOC) [file pone.0053137.s001.doc]

(**a**)

(**b**)

(**c**)

***F*_cd_ (%)**

***F*_ncd_ (%)**

***F*_o_ (%)**

**Median = 1.6**

**Median = 1.4**

**Median = 2.2**

**Figure S1.** Distribution of G4 abundance scores (the *F* scores) and corresponding cumulative frequencies (the *CF* scores) on coding strand (**a**), template strand (**b**), and both strands (**c**). In the analysis, TRRs from 46,205 transcripts exported from ENSEMBL with redundancy were included. TRRs of transcripts without G4-forming motifs were excluded in calculating the distribution and corresponding cumulative frequency.
