# Supplementary material for: Possible Regulatory Roles of Promoter G-Quadruplexes in Cardiac Function-Related Genes – Human TnIc as a Model
Source: PLoS One. 2013 Jan 9;8(1):e53137. doi: 10.1371/journal.pone.0053137 (PMC3541360; doi:10.1371/journal.pone.0053137)
Supplement: Figure S2 — Probability distribution and location significance scores (the Q scores) distribution of G4s in TRRs of transcripts. (a) Frequency of PQS (potential quadruplex sequence) at each position (Wk as described in Equation 3) on coding strand (black), template strand (red), and both strands (green) in TRRs of all transcripts. TRRs from 46,205 transcripts exported from ENSEMBL with redundancy were included. (b∼d) Distribution of G4 location significance (lower graphs) and corresponding cumulative frequency (upper graphs) of all transcripts available in ENSEMBL database on the coding strands, template strand, and both strands, respectively. TRRs of transcripts without G4-forming motifs were excluded in calculating the distribution and corresponding cumulative frequency. (DOC) [file pone.0053137.s002.doc]

(**a**)

(**b**)

(**c**)

(**d**)

***Q*_cd_ (%)**

***Q*_ncd_ (%)**

***Q*_o_ (%)**

**Median = 2.5**

**Median = 1.7**

**Median = 3.2**

**Coding Strand**

**Template Strand**

**Both Strand**

**Figure S2.** Probability distribution and location significance scores (the *Q* scores) distribution of G4s in TRRs of transcripts. (**a**) Frequency of PQS (potential quadruplex sequence) at each position (*W_k_* as described in Equation 3) on coding strand (black), template strand (red), and both strands (green) in TRRs of all transcripts. TRRs from 46,205 transcripts exported from ENSEMBL with redundancy were included. (**b ~ d**) Distribution of G4 location significance (lower graphs) and corresponding cumulative frequency (upper graphs) of all transcripts available in ENSEMBL database on the coding strands, template strand, and both strands, respectively. TRRs of transcripts without G4-forming motifs were excluded in calculating the distribution and corresponding cumulative frequency.
