# Supplementary material for: Possible Regulatory Roles of Promoter G-Quadruplexes in Cardiac Function-Related Genes – Human TnIc as a Model
Source: PLoS One. 2013 Jan 9;8(1):e53137. doi: 10.1371/journal.pone.0053137 (PMC3541360; doi:10.1371/journal.pone.0053137)
Supplement: Figure S3 — Correlation between the location significance (the CQ score) and abundance (the CF socre) of G4s identified in TRRs of all transcripts with redundancy. (a∼c) Correlation between G4 location significance and G4 abundance on the coding strand (CQ cd vs. CF cd), template strand (CQ ncd vs. CF ncd), and both strands (CQ o vs. CF o), respectively. In each map, transcripts with the TRRs CQ and CF scores larger than 50% are identified as G4-important transcripts, and those with both values smaller than 50% are G4-less-important (or less G4-important) transcripts. Correlation coefficients between CF and CQ on coding strand (CF cd vs. CQ cd), template strand (CF ncd vs. CQ ncd), and both strands (CF o vs. CQ o) are 0.57, 0.50, and 0.61, respectively. However, corresponding correlation coefficients for G4-important TRRs are 0.61 (coding strand), 0.58 (template strand), and 0.66 (both strands), respectively, while those for G4-less important TRRs are 0.10, 0.09, and 0.34 respectively. Apparently, in G4-important TRRs, their G4 abundance is more positively correlated to their G4 location significance. (DOC) [file pone.0053137.s003.doc]

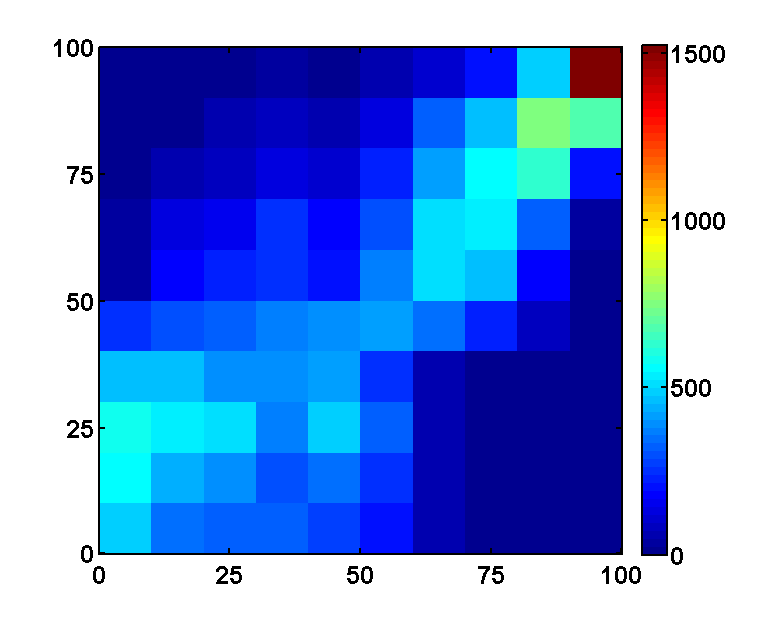

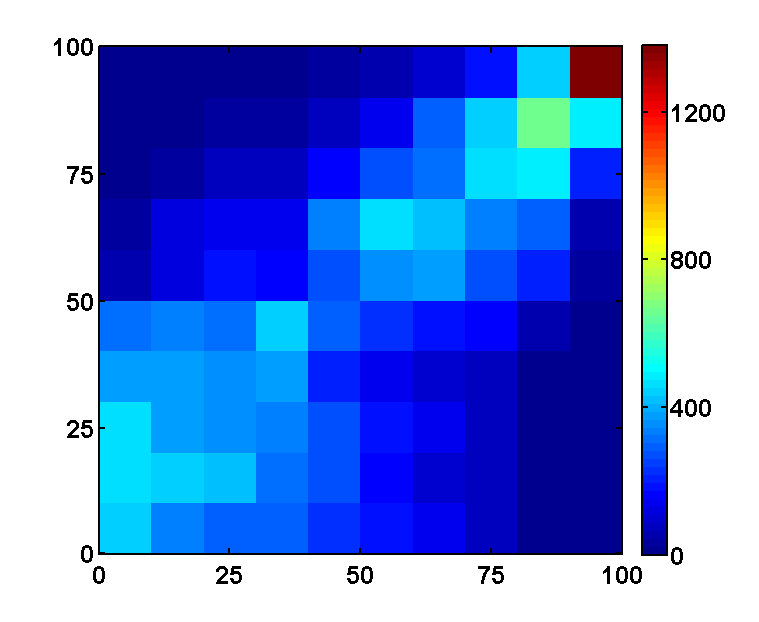

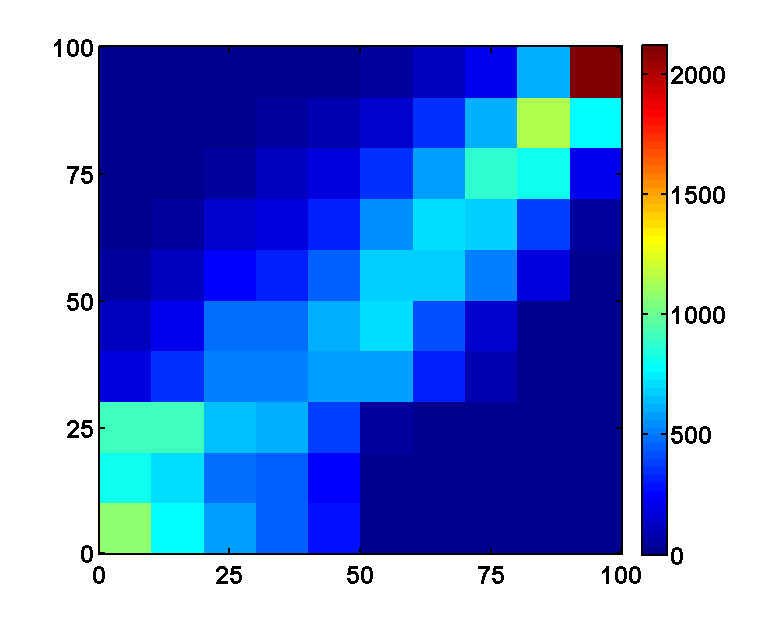


(**a**)

(**b**)

(**c**)

***CQ*_cd_ (%)**

***CF*_cd_ (%)**

***CQ*_ncd_ (%)**

***CF*_ncd_ (%)**

***CQ*_o_ (%)**

***CF*_o_ (%)**

**Figure S3.** Correlation between the location significance (the *CQ* score) and abundance (the *CF* socre) of G4s identified in TRRs of all transcripts with redundancy. (**a ~ c**) Correlation between G4 location significance and G4 abundance on the coding strand (*CQ*_cd_ vs. *CF*_cd_), template strand (*CQ*_ncd_ vs. *CF*_ncd_), and both strands (*CQ*_o_ vs. *CF*_o_), respectively. In each map, transcripts with the TRRs *CQ* and *CF* scores larger than 50 % are identified as G4-important transcripts, and those with both values smaller than 50 % are G4-less-important (or less G4-important) transcripts. Correlation coefficients between *CF* and *CQ* on coding strand (*CF*_cd_ vs. *CQ*_cd_), template strand (*CF*_ncd_ vs. *CQ*_ncd_), and both strands (*CF*_o_ vs. *CQ*_o_) are 0.57, 0.50, and 0.61, respectively. However, corresponding correlation coefficients for G4-important TRRs are 0.61 (coding strand), 0.58 (template strand), and 0.66 (both strands), respectively, while those for G4-less important TRRs are 0.10, 0.09, and 0.34 respectively. Apparently, in G4-important TRRs, their G4 abundance is more positively correlated to their G4 location significance.
