# Supplementary material for: Possible Regulatory Roles of Promoter G-Quadruplexes in Cardiac Function-Related Genes – Human TnIc as a Model
Source: PLoS One. 2013 Jan 9;8(1):e53137. doi: 10.1371/journal.pone.0053137 (PMC3541360; doi:10.1371/journal.pone.0053137)
Supplement: Figure S4 — Native and denaturing gel electrophoresis indicating the formation of G4s. (a) TnIc MNSG4, 1 repeat: Lane1 and 2 are oligos TrMNS-II (C-rich) and TrMNS-I (G-rich) run in a native gel. Lane 3 and 4 are oligos run in a denaturing gel as controls. (b) TnIc −80 G4: Lane1 and 2 are oligos Tr-80-II (C-rich) and Tr-80-I (G-rich) run in a native gel. Lane 3 and 4 are oligos run in a denaturing gel as controls. Formation of intramolecular G4s is proved by species with fast mobility under native conditions (indicated by black arrows) but not under denaturing conditions. Intermolecular G4s formed by the −80 G4 forming sequence are indicated by gray arrows in (b). (DOC) [file pone.0053137.s004.doc]

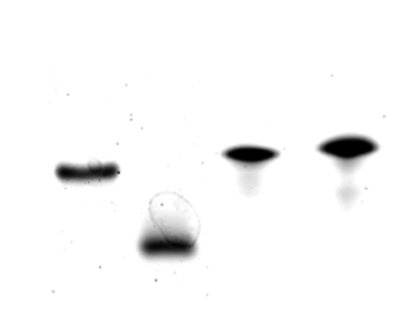


**1 2 3 4**

(**a**)


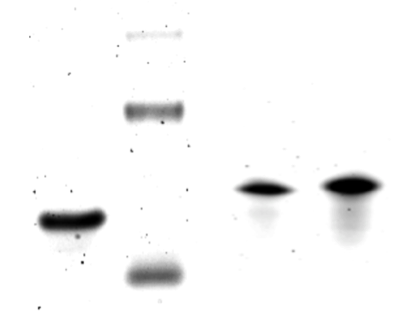


**1 2 3 4**

(**b**)

**Figure S4.** Native and denaturing gel electrophoresis indicating the formation of G4s. (**a**) TnIc MNSG4, 1 repeat: Lane1 and 2 are oligos **Tr_MNS_-II** (C-rich) and **Tr_MNS_-I** (G-rich) run in a native gel. Lane 3 and 4 are oligos run in a denaturing gel as controls. (**b**) TnIc -80G4: Lane1 and 2 are oligos **Tr_-80_-II** (C-rich) and **Tr_-80_-I** (G-rich) run in a native gel. Lane 3 and 4 are oligos run in a denaturing gel as controls. Formation of intramolecular G4s is proved by species with fast mobility under native conditions (indicated by black arrows) but not under denaturing conditions. Intermolecular G4s formed by the -80G4 forming sequence are indicated by gray arrows in (**b**).
