# Supplementary material for: Possible Regulatory Roles of Promoter G-Quadruplexes in Cardiac Function-Related Genes – Human TnIc as a Model
Source: PLoS One. 2013 Jan 9;8(1):e53137. doi: 10.1371/journal.pone.0053137 (PMC3541360; doi:10.1371/journal.pone.0053137)
Supplement: Figure S5 — DMS footprinting results of TnIc MNSG4 and −80 G4. (a) DMS footprinting of MNSG4 (oligo TrMNS-I). Strong DMS protection is observed at G1∼G3, G5∼G7, G11∼G13, and G14∼G15. Two guanines at the 3′ of MNSG4 (G17∼18, oligo TrMNS-I) are not involved in G4 formation. Compared to other guanines involved in loops (G4, G8, and G9), G10 is partially protected from DMS. Because G13 is fully protected from DMS, this partial protection of G10 may be due to the gyration hindrance from the adjacent G4 structure, which blocks its access by DMS. (b) DMS footprinting of −80 G4 (oligo TrMNS-I). In the first 5′ run of guanines (G1∼G5), partial protection of guanines is apparent. Meanwhile, in the second and third runs of guanines (G9∼G12 and G13∼G16), partially protected flanking guanines in each G-run are also found (G9/G12 and G12/G16) with two fully protected guanines in the middle (G10∼G11 and G14∼G15). This poorly defined footprinting pattern of −80 G4 element is possibly due to the long first loop (at least 7 nucleotides) which induces flexibility of the G4 conformations. Together with CD experiments, it’s highly likely that this sequence can fold into a mixture of exchanging parallel intramolecular G4s with different loop arrangements in KCl solution. Furthermore, in the second and third G-runs, it is clear that G12 and G13 are better protected from DMS than G9 and G16, which may reflect the preference of different intramolecular G4s. (DOC) [file pone.0053137.s005.doc]

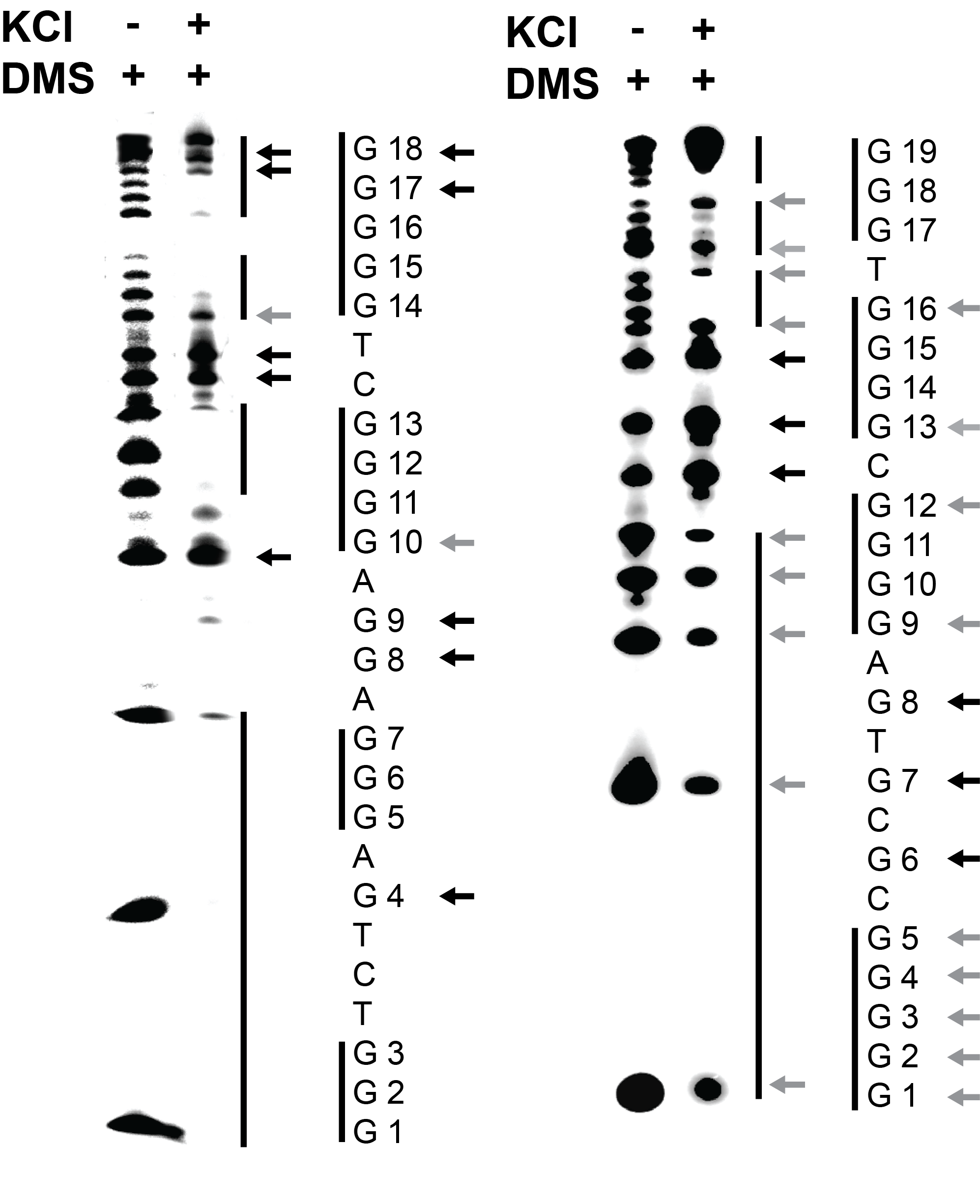


(**a**)

(**b**)

**Figure S5.** DMS footprinting results of TnIc MNSG4 and -80G4. (**a**) DMS footprinting of MNSG4 (oligo **Tr_MNS_-I**). Strong DMS protection is observed at G1~G3, G5~G7, G11~G13, and G14~G15. Two guanines at the 3’ of MNSG4 (G17~18, oligo **Tr_MNS_-I**) are not involved in G4 formation. Compared to other guanines involved in loops (G4, G8, and G9), G10 is partially protected from DMS. Because G13 is fully protected from DMS, this partial protection of G10 may be due to the gyration hindrance from the adjacent G4 structure, which blocks its access by DMS. (**b**) DMS footprinting of -80G4 (oligo **Tr_MNS_-I**). In the first 5’ run of guanines (G1 ~ G5), partial protection of guanines is apparent. Meanwhile, in the second and third runs of guanines (G9~G12 and G13~G16), partially protected flanking guanines in each G-run are also found (G9/G12 and G12/G16) with two fully protected guanines in the middle (G10~G11 and G14~G15). This poorly defined footprinting pattern of -80G4 element is possibly due to the long first loop (at least 7 nucleotides) which induces flexibility of the G4 conformations. Together with CD experiments, it’s highly likely that this sequence can fold into a mixture of exchanging parallel intramolecular G4s with different loop arrangements in KCl solution. Furthermore, in the second and third G-runs, it is clear that G12 and G13 are better protected from DMS than G9 and G16, which may reflect the preference of different intramolecular G4s.
