# Supplementary figures and images for: Possible Regulatory Roles of Promoter G-Quadruplexes in Cardiac Function-Related Genes – Human TnIc as a Model
Source: PLoS One. 2013 Jan 9;8(1):e53137. doi: 10.1371/journal.pone.0053137 (PMC3541360; doi:10.1371/journal.pone.0053137)

(**a**)


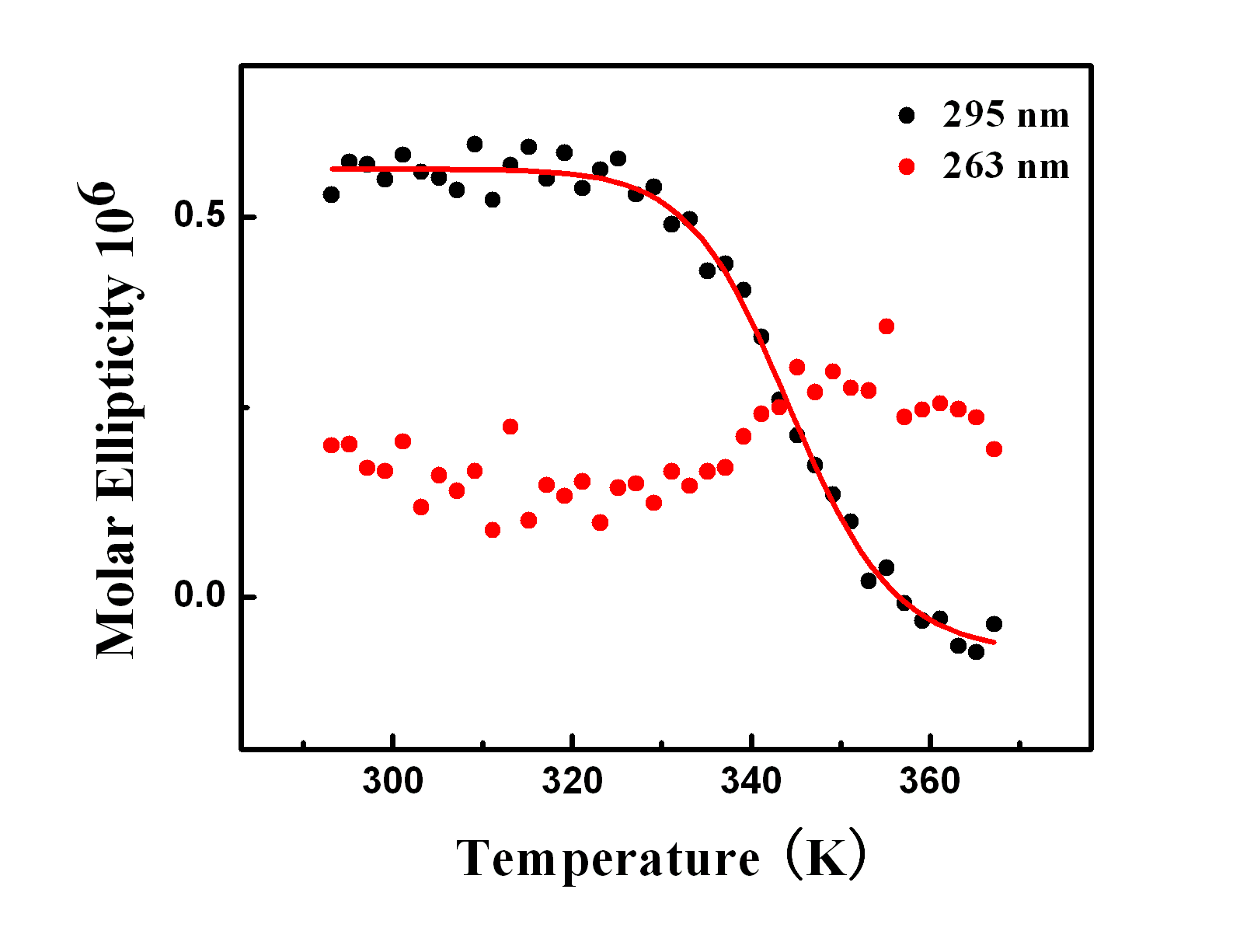


(**b**)


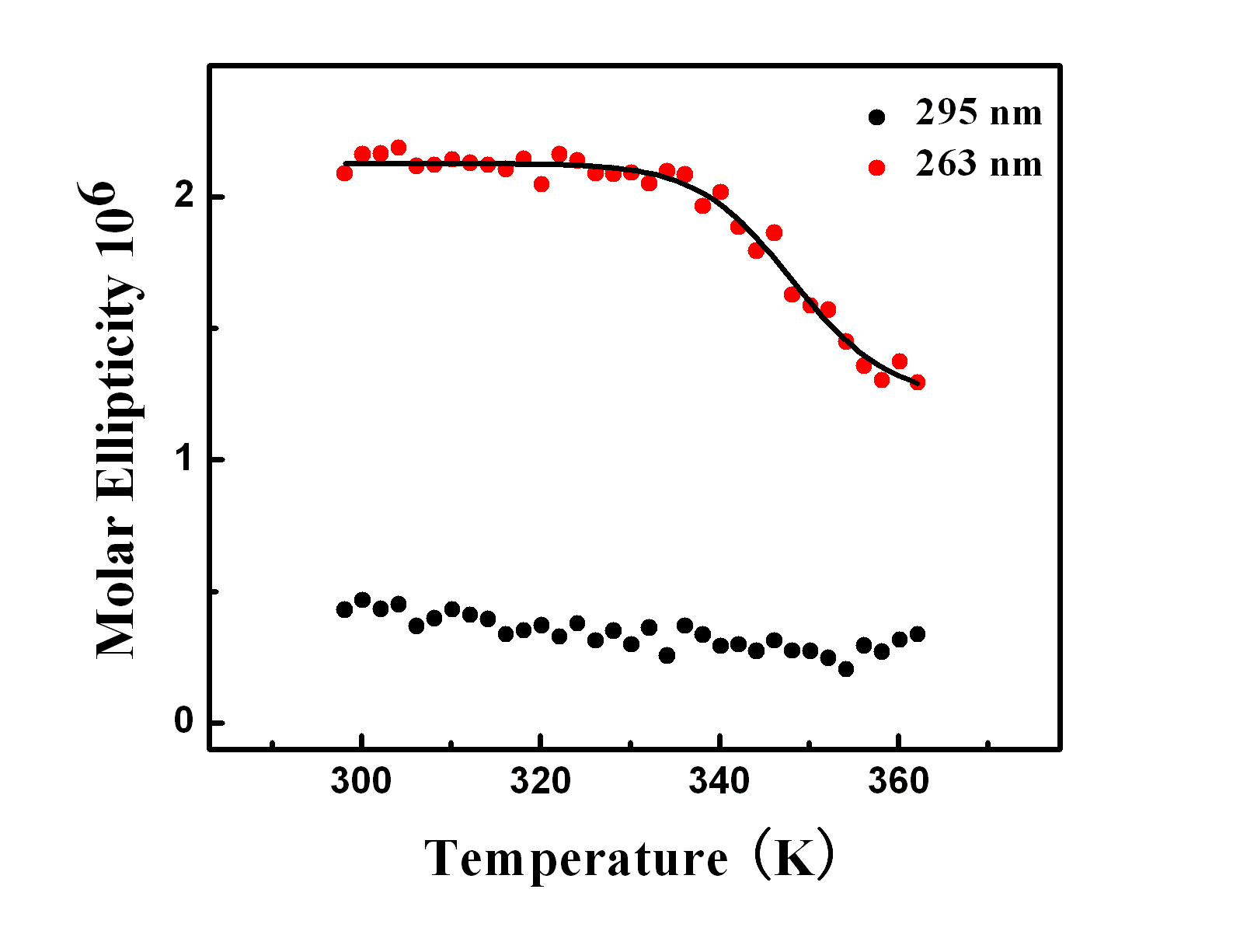


**Figure S6.** CD melting results of (**a**) single-repeat MNSG4, and (**b**) -80G4.

Supplement: Figure S6 — CD melting of the two promoter G-quadruplex sequences. (a) single-repeat MNSG4. (b) −80 G4. (DOC) [file pone.0053137.s006.doc]
