# Supplementary material for: Possible Regulatory Roles of Promoter G-Quadruplexes in Cardiac Function-Related Genes – Human TnIc as a Model
Source: PLoS One. 2013 Jan 9;8(1):e53137. doi: 10.1371/journal.pone.0053137 (PMC3541360; doi:10.1371/journal.pone.0053137)
Supplement: Figure S7 — Arrhenius plot for unfolding of TnIc G4s. (a) Arrhenius plot for the slow decaying component identified in the unfolding process of TnIc MNSG4. Empty circles represent opening rates measured at different temperatures, and red line is the result fitted by Arrhenius equation. The activation energy is determined to be 22.1±0.4 kJ mol−1. (b) Arrhenius plot for the slow and fast decaying components identified in the unfolding process of TnIc −80 G4. Empty triangles and squares represent opening rates of the fast and slow decaying components, respectively, and red lines are results fitted by Arrhenius equation. The activation energies for the fast and slow decaying components are 86.1±23.7 and 100.6±6.6 kJ mol−1, respectively, as determined by the fits. (DOC) [file pone.0053137.s007.doc]

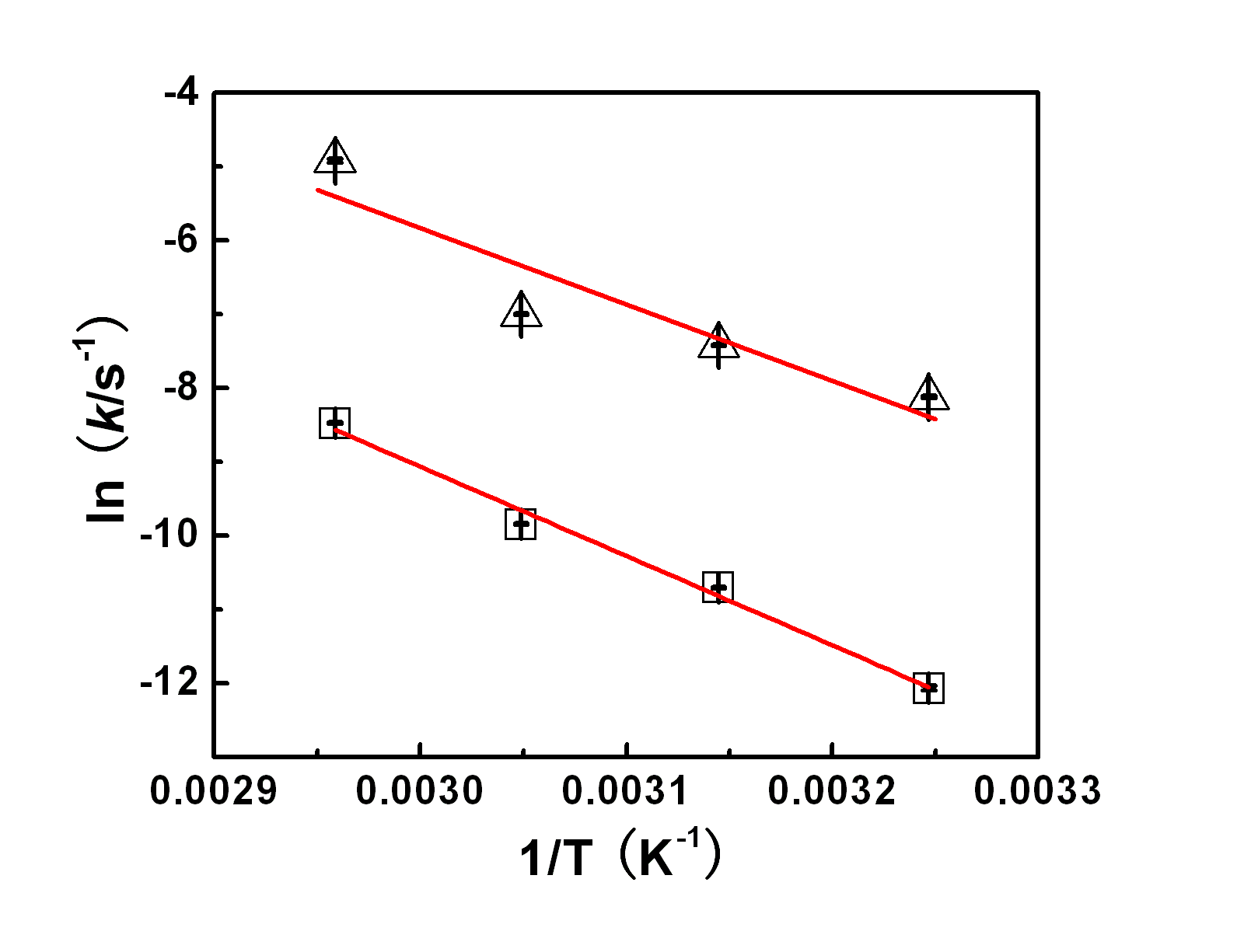

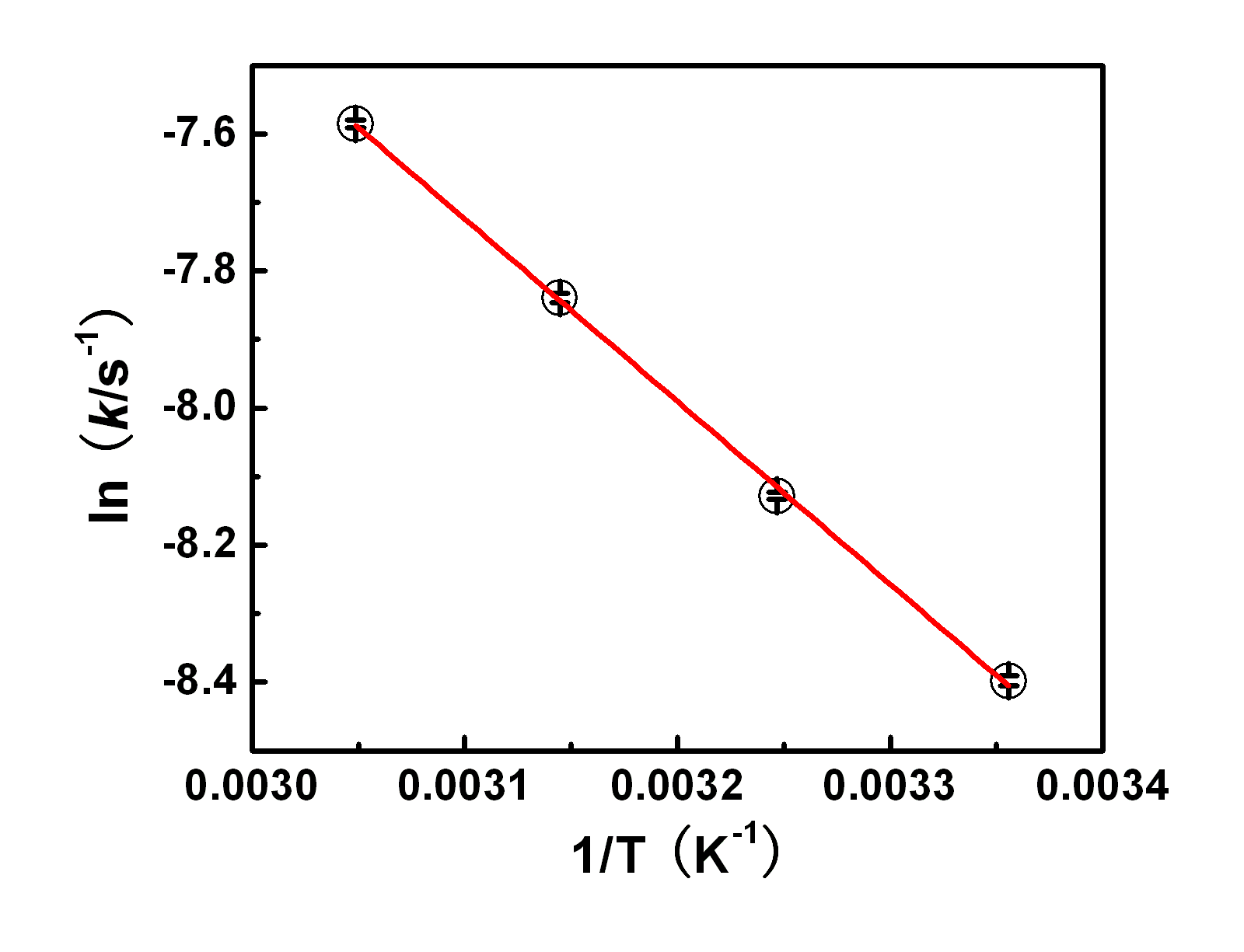


(**a**)

(**b**)

**Figure S7.** Arrhenius plot for unfolding of TnIc G4s.

(**a**) Arrhenius plot for the slow decaying component identified in the unfolding process of TnIc MNSG4. Empty circles represent opening rates measured at different temperatures, and red line is the result fitted by Arrhenius equation. The activation energy is determined to be 22.1 ± 0.4 kJ mol^-1^. (**b**) Arrhenius plot for the slow and fast decaying components identified in the unfolding process of TnIc -80G4. Empty triangles and squares represent opening rates of the fast and slow decaying components, respectively, and red lines are results fitted by Arrhenius equation. The activation energies for the fast and slow decaying components are 86.1 ± 23.7 and 100.6 ± 6.6 kJ mol^-1^, respectively, as determined by the fits.
