# Supplementary material for: Possible Regulatory Roles of Promoter G-Quadruplexes in Cardiac Function-Related Genes – Human TnIc as a Model
Source: PLoS One. 2013 Jan 9;8(1):e53137. doi: 10.1371/journal.pone.0053137 (PMC3541360; doi:10.1371/journal.pone.0053137)
Supplement: Figure S8 — CD melting of TnIc MNSG4 and −80 G4 in the presence of the G4-binding ligand (complex 3). (a) CD melting spectra of TnIc MNSG4 (oligo TrMNS-I, 5 µM) in the presence of 10 µM complex 3. (b) CD signal changes of TnIc MNSG4 (oligo TrMNS-I) at 295 nm (black dots) and 263 (red dots) nm wavelengths in response to temperature changes, both of which were fitted by a sigmoidal model (indicated by red line and black line respectively). The melting temperature measured at 265 nm and 295 nm are 90.4±0.7°C and 86.2±2.1°C respectively (c) CD melting spectra of TnIc −80 G4 (oligo Tr-80-I, 5 µM) in the presence of 10 µM complex 3. (d) CD signal changes of TnIc −80 G4 (oligo Tr-80-I) at 295 nm (black dots) and 263 (red dots) nm wavelengths in response to temperature changes, both of which were fitted by a sigmoidal model (indicated by red line and black line respectively). All experiments were carried out in 10 mM Tris-HCl buffer (pH 7.4) containing 100 mM K+. (DOC) [file pone.0053137.s008.doc]

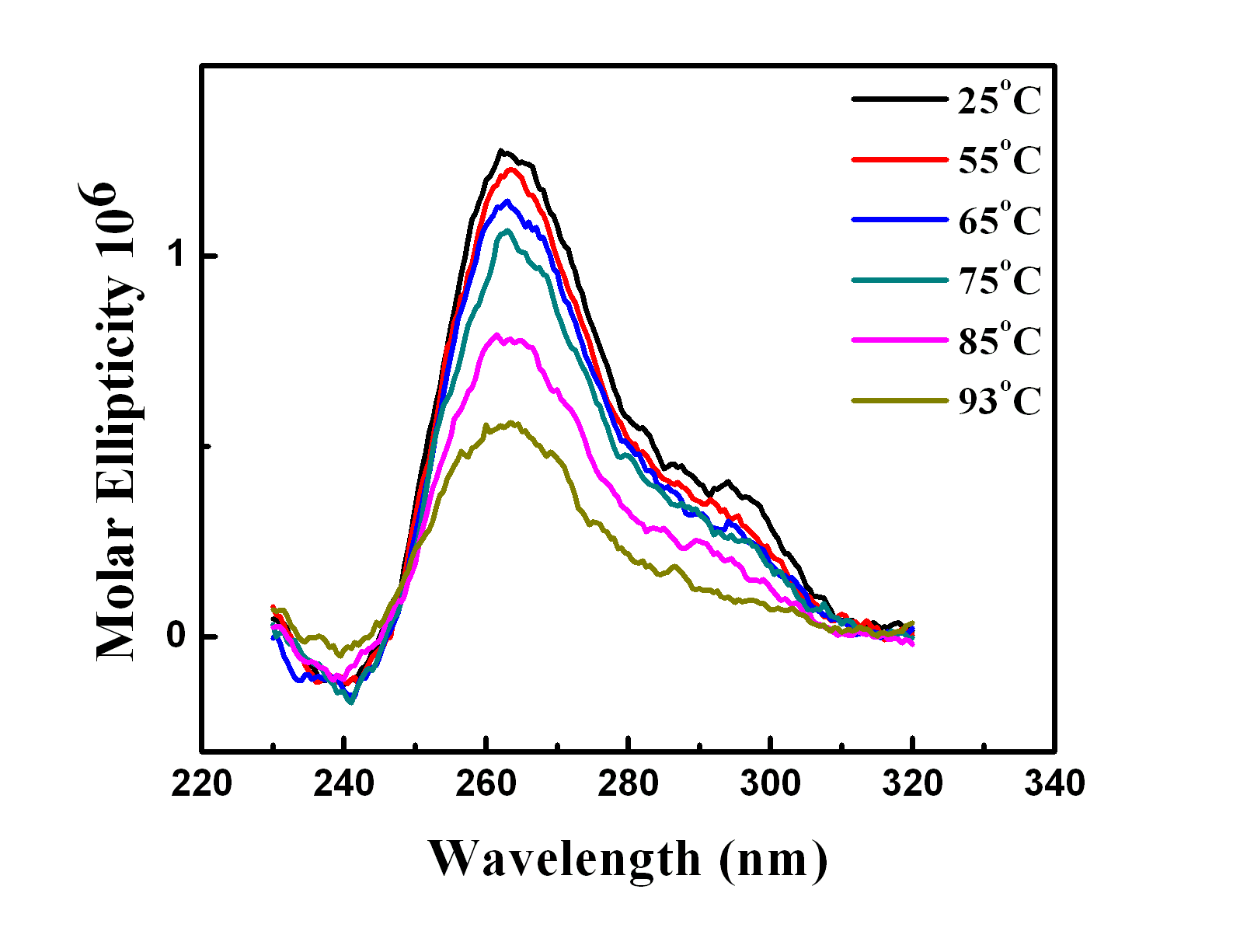

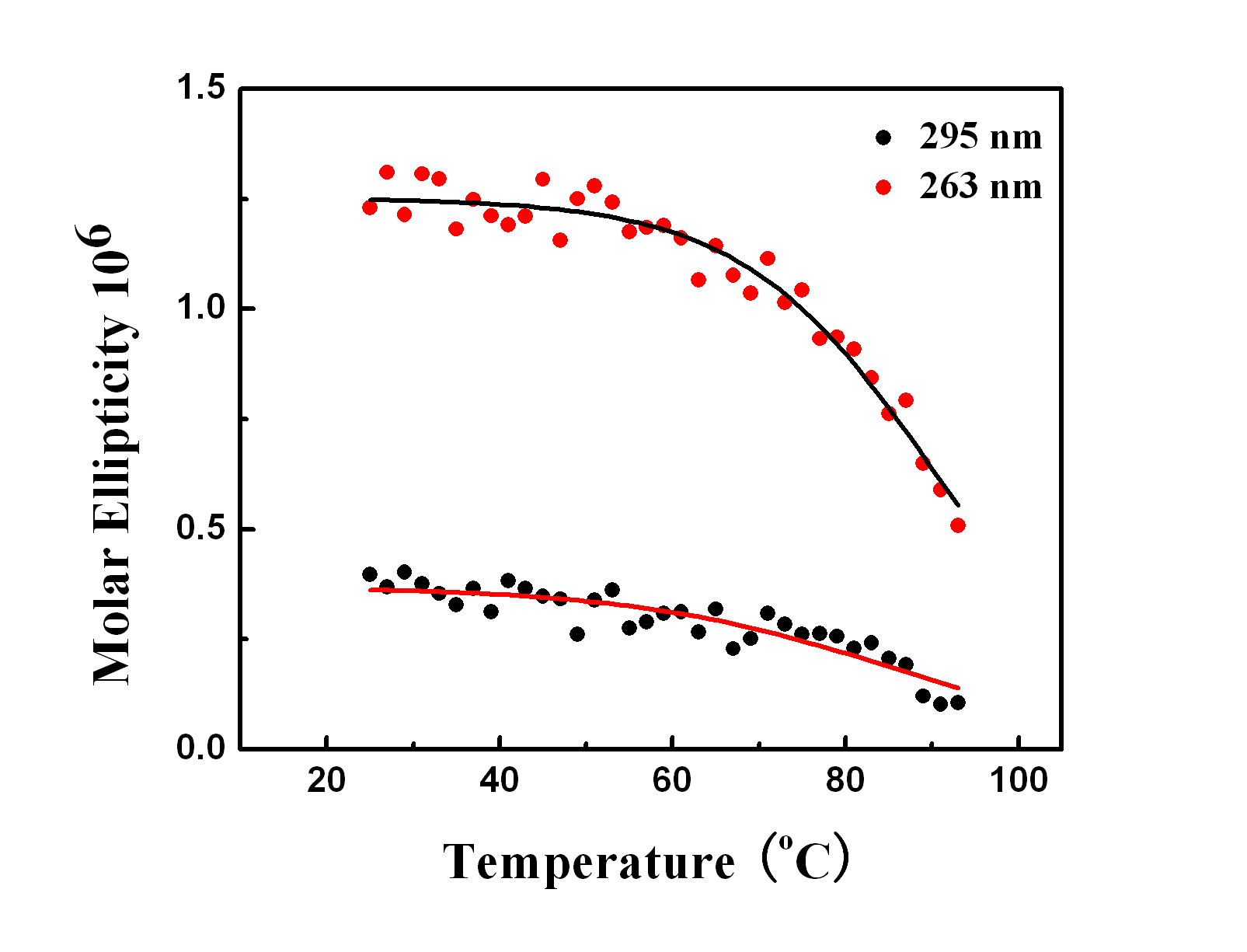

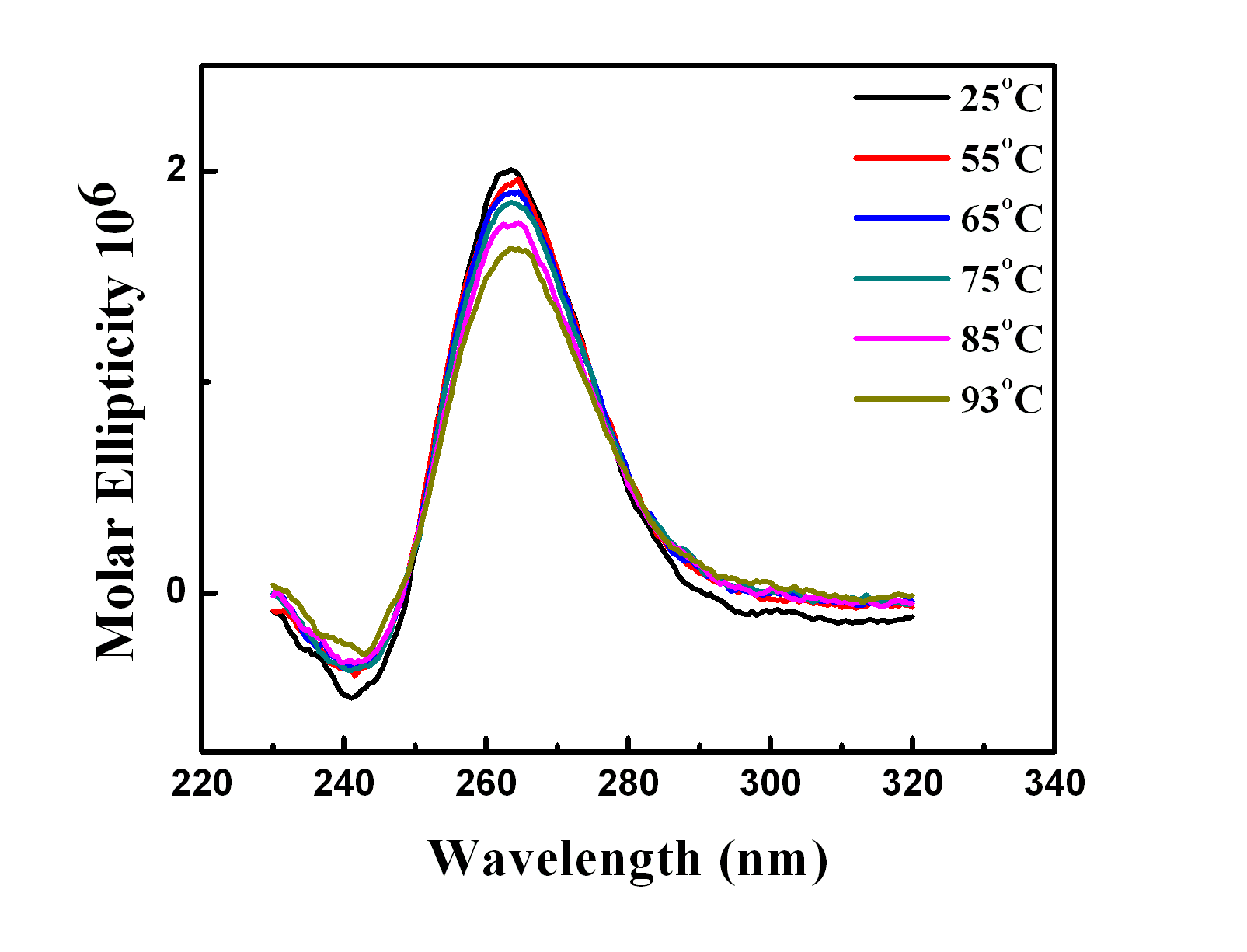

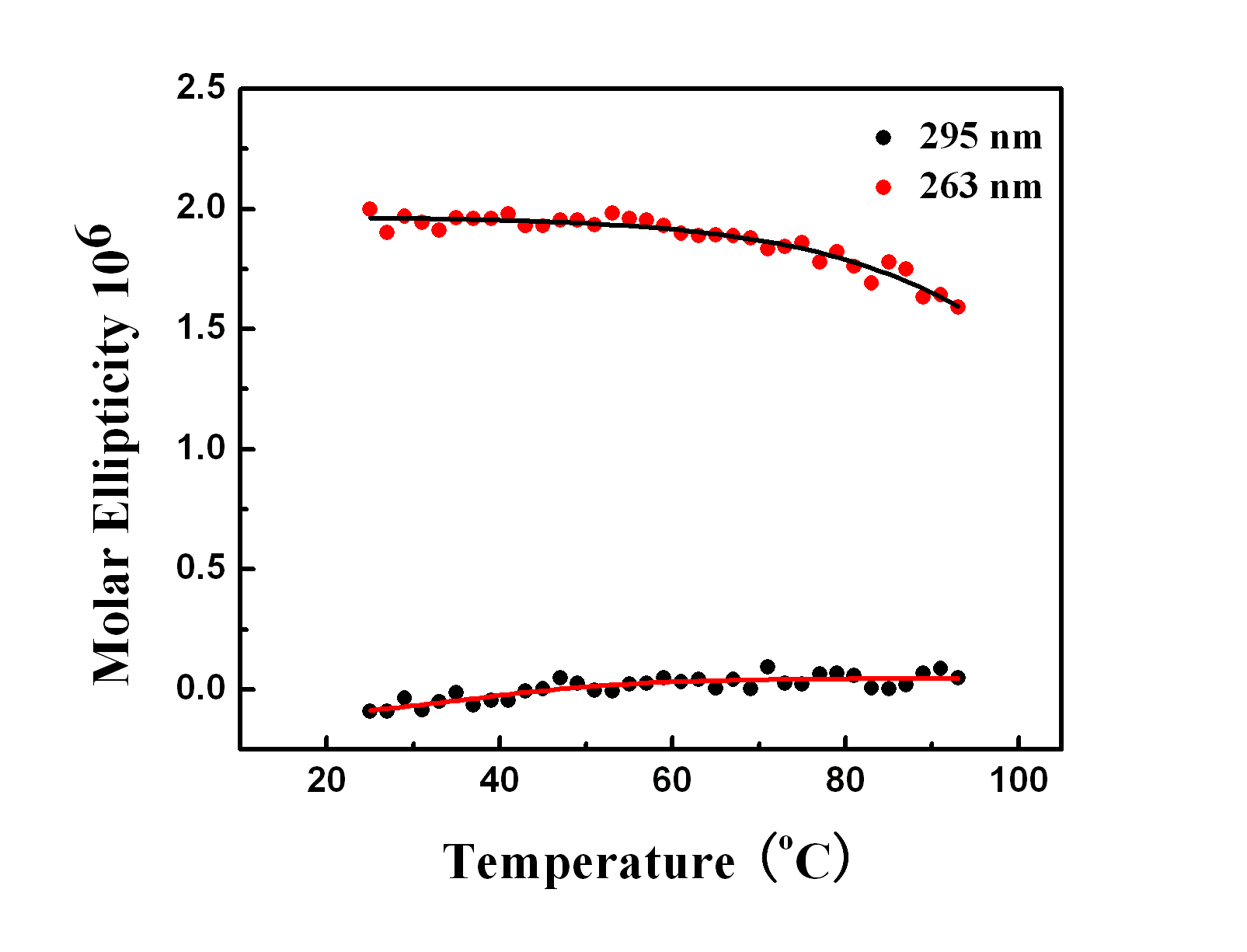


**a**

**b**

**c**

**d**

**Figure S8.** CD melting of TnIc MNSG4 and -80G4 in the presence of the G4-binding ligand (complex **3)**. (**a**) CD melting spectra of TnIc MNSG4 (oligo **Tr_MNS_-I**, 5 μM**)** in the presence of 10 μM complex **3**. (**b**) CD signal changes of TnIc MNSG4 (oligo **Tr_MNS_-I)** at 295 nm (black dots) and 263 (red dots) nm wavelengths in response to temperature changes, both of which were fitted by a sigmoidal model (indicated by red line and black line respectively). The melting temperature measured at 265 nm and 295 nm are 90.4 ± 0.7 °C and 86.2 ± 2.1 °C respectively (**c**) CD melting spectra of TnIc -80G4 (oligo **Tr_-80_-I**, 5 μM**)** in the presence of 10 μM complex **3**. (**d**) CD signal changes of TnIc -80G4 (oligo **Tr_-80_-I)** at 295 nm (black dots) and 263 (red dots) nm wavelengths in response to temperature changes, both of which were fitted by a sigmoidal model (indicated by red line and black line respectively). All experiments were carried out in 10 mM Tris-HCl buffer (pH 7.4) containing 100mM K^+^.
