# Supplementary material for: Possible Regulatory Roles of Promoter G-Quadruplexes in Cardiac Function-Related Genes – Human TnIc as a Model
Source: PLoS One. 2013 Jan 9;8(1):e53137. doi: 10.1371/journal.pone.0053137 (PMC3541360; doi:10.1371/journal.pone.0053137)
Supplement: Figure S9 — Comparison of the transcription activities of human TnIc promoters containing variable number of MNSG4. Transcription activities of human TnIc promoters containing 6 repeats of consensus MNSG4 (hTnIc-6MNS(E)-WT), 3 repeats of consensus MNSG4 (hTnIc-3MNS(E)-WT), 1 repeat of consensus MNSG4 (hTnIc-1MNS(E)-WT), and no repeat of MNSG4 (hTnIc-299-WT). No transcription activity difference was found among these human TnIc promoters (P≥0.05, no significant differences). (DOC) [file pone.0053137.s009.doc]

**
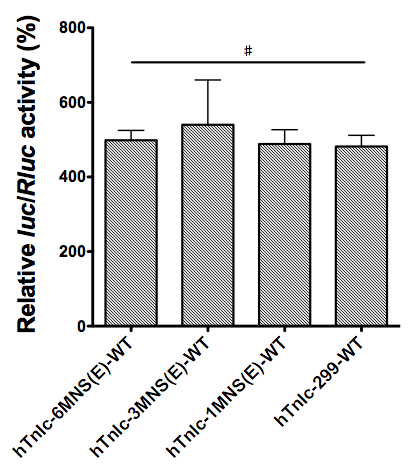
**

**Figure S9.** Comparison of the transcription activities of human *TnIc* promoters containing variable number of MNSG4. Transcription activities of human *TnIc* promoters containing 6 repeats of consensus MNSG4 (hTnIc-6MNS(E)-WT), 3 repeats of consensus MNSG4 (hTnIc-3MNS(E)-WT), 1 repeat of consensus MNSG4 (hTnIc-1MNS(E)-WT), and no repeat of MNSG4 (hTnIc-299-WT). No transcription activity difference was found among these human *TnIc* promoters (*P* ≥ 0.05, no significant differences).
