# Supplementary material for: Possible Regulatory Roles of Promoter G-Quadruplexes in Cardiac Function-Related Genes – Human TnIc as a Model
Source: PLoS One. 2013 Jan 9;8(1):e53137. doi: 10.1371/journal.pone.0053137 (PMC3541360; doi:10.1371/journal.pone.0053137)
Supplement: Figure S10 — Transcription activities of human TnIc promoters with wild type and mutated −80 G4. Mutation of −80 G4 alone (hTnIc-80 G4M) did not change the transcription activity, while the mutation of Sp1 binding site (hTnIc-80sp1M-1/2) led to∼25% depression in the transcription activity. When both G4 formation and Sp1 binding sites were mutated (hTnIc-80 G4/sp1M), 50% decrease of the transcription activity was found (* P<0.05 and ♯ P≥0.05 no significant differences). (DOC) [file pone.0053137.s010.doc]

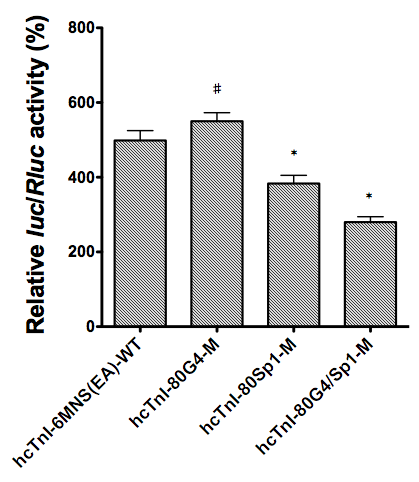


**Figure S10.** Transcription activities of human *TnIc* promoters with wild type and mutated -80G4. Mutation of -80G4 alone (hTnIc-80G4M) did not change the transcription activity, while the mutation of Sp1 binding site (hTnIc-80sp1M-1/2) led to ~ 25% depression in the transcription activity. When both G4 formation and Sp1 binding sites were mutated (hTnIc-80G4/sp1M), 50% decrease of the transcription activity was found (* *P* < 0.05 and ♯ *P* ≥ 0.05 no significant differences).
