# Supplementary material for: Possible Regulatory Roles of Promoter G-Quadruplexes in Cardiac Function-Related Genes – Human TnIc as a Model
Source: PLoS One. 2013 Jan 9;8(1):e53137. doi: 10.1371/journal.pone.0053137 (PMC3541360; doi:10.1371/journal.pone.0053137)
Supplement: Table S1 — The rankings and scores of G4 enrichment in TRRs of genes active in different tissues. G4s in the full-length TRR (Whole TRR), distal promoter region (−2,000∼−501 bp), proximal promoter region (−500∼−1 bp), and downstream region (TSS∼+1,000 bp) were searched and the procedure was repeated randomly for five times. (DOC) [file pone.0053137.s011.doc]

**Table S1.** The rankings and scores of G4 enrichment in TRRs of genes active in different tissues. G4s in the full-length TRR (Whole TRR), distal promoter region (-2,000 ~ -501 bp), proximal promoter region (-500 ~ -1 bp), and downstream region (TSS ~ +1,000 bp) were searched and the procedure was repeated randomly for five times.

| **Tissues** | **The Ranking* (descending) and Score (%) of G4 Enrichment in Different Tissues** | | | |
| --- | --- | --- | --- | --- |
|  | **Whole TRR** | **-2,000 ~ -501 bp** | **-500 ~ -1 bp** | **TSS ~ +1,000 bp** |
| Lung | 1 82.8 ± 0.4 | 2 40.0 ± 3.0 | 1 49.7 ± 0.3 | 3 63.2 ± 1.8 |
| Whole Brain | 2 81.9 ± 0.1 | 6 34.1 ± 0.6 | 9 45.7 ± 0.4 | 1 64.1 ± 0.2 |
| Caudate Nucleus | 3 80.8 ± 0.2 | 14 32.9 ± 0.5 | 6 46.3 ± 0.4 | 4 62.7 ± 0.4 |
| Heart | 4 80.0 ± 0.2 | 1 40.4 ± 0.2 | 11 45.2 ± 1.0 | 9 60.2 ± 1.0 |
| Cerebellum Peduncles | 5 79.9 ± 0.6 | 4 36.4 ± 0.2 | 8 46.1 ± 0.6 | 2 63.8 ± 0.6 |
| Amygdala | 6 79.8 ± 0.3 | 23 31.1 ± 0.1 | 14 44.2 ± 0.4 | 5 62.31 ± 0.04 |
| Thalamus | 7 79.5 ± 0.4 | 22 31.3 ± 0.2 | 18 43.1 ± 2.5 | 6 62.2 ± 0.7 |
| Prostate | 8 79.4 ± 0.9 | 9 33.9 ± 1.2 | 17 43.1 ± 1.1 | 17 58.2 ± 1.8 |
| Cerebellum | 9 79.2 ± 0.3 | 7 34.1 ± 0.3 | 19 42.8 ± 0.8 | 7 61.42 ± 0.02 |
| Adrenal Gland | 10 78.8 ± 1.6 | 18 32.4 ± 0.6 | 7 46.1 ± 0.8 | 22 56.8 ± 2.2 |
| Adipose Tissue | 11 78.7 ± 0.1 | 8 34.0 ± 2.3 | 5 46.8 ± 1.2 | 19 57.7 ± 0.2 |
| Olfactory Bulb | 12 78.5 ± 0.5 | 3 36.7 ± 0.7 | 3 49.2 ± 1.8 | 14 59.5 ± 1.5 |
| Cingulate Cortex | 13 78.2 ± 0.2 | 16 32.52 ± 0.03 | 20 42.6 ± 0.1 | 13 59.6 ± 0.1 |
| Temporal Lobe | 14 77.8 ± 0.2 | 17 32.5 ± 0.3 | 10 45.31 ± 0.04 | 10 60.0 ± 0.2 |
| Prefrontal Cortex | 15 77.7 ± 0.1 | 20 31.5 ± 0.2 | 15 43.4 ± 0.2 | 8 61.0 ± 0.1 |
| Placenta | 16 77.6 ± 0.4 | 10 33.3 ± 0.9 | 12 45.0 ± 0.6 | 16 58.8 ± 0.3 |
| Hypothalamus | 17 77.1 ± 0.2 | 31 29.6 ± 0.2 | 25 41.2 ± 0.3 | 12 59.6 ± 0.3 |
| Spinal Cord | 18 76.7 ± 1.3 | 26 30.8 ± 1.0 | 13 44.5 ± 1.5 | 11 59.9 ± 0.7 |
| Occipital Lobe | 19 76.3 ± 1.0 | 28 30.1 ± 0.4 | 23 41.6 ± 0.7 | 15 59.0 ± 0.5 |
| Fetal Thyroid | 20 76.0 ± 0.9 | 27 30.3 ± 0.7 | 2 49.2 ± 2.8 | 34 51.5 ± 1.2 |
| Parietal Lobe | 21 75.72 ± 0.03 | 24 31.03 ± 0.03 | 24 41.4 ± 0.4 | 21 56.9 ± 0.1 |
| Thyroid | 22 75.6 ± 0.8 | 11 33.3 ± 0.5 | 16 43.3 ± 0.5 | 23 56.3 ± 0.2 |
| Medulla Oblongata | 23 75.6 ± 0.1 | 30 29.7 ± 0.5 | 22 41.9 ± 0.8 | 18 57.9 ± 0.2 |
| Kidney | 24 75.4 ± 0.1 | 25 30.9 ± 0.4 | 26 41.1 ± 0.3 | 27 54.5 ± 0.2 |
| Subthalamic Nucleus | 25 75.0 ± 0.1 | 21 31.3 ± 0.4 | 28 39.8 ± 0.3 | 20 57.6 ± 0.1 |
| Liver | 26 74.9 ± 0.2 | 19 31.9 ± 0.9 | 27 40.4 ± 0.1 | 29 54.3 ± 0.2 |
| Tumor | 27 74.5 ± 0.4 | 39 28.0 ± 1.5 | 37 35.8 ± 1.4 | 32 54.2 ± 0.9 |

*(continued Table S1)*

| **Tissues** | **The Ranking* (descending) and Score (%) of G4 Enrichment in Different Tissues** | | | |
| --- | --- | --- | --- | --- |
|  | **Whole TRR** | **-2,000 ~ -501 bp** | **-500 ~ -1 bp** | **TSS ~ +1,000 bp** |
| White Blood Cells | 28 73.3 ± 0.3 | 35 29.1 ± 0.4 | 31 38.9 ± 0.8 | 25 55.0 ± 0.1 |
| Smooth Muscle | 29 73.1 ± 1.5 | 13 33.1 ± 3.0 | 4 48.6 ± 2.3 | 28 54.4 ± 0.6 |
| Pons | 30 72.9 ± 0.2 | 29 29.9 ± 0.1 | 30 39.0 ± 0.1 | 31 54.3 ± 0.2 |
| Globus Pallidus | 31 72.5 ± 0.2 | 32 29.5 ± 0.1 | 33 38.48 ± 0.04 | 30 54.3 ± 0.1 |
| Bone Marrow | 32 72.1 ± 0.7 | 15 32.6 ± 0.8 | 48 33.5 ± 1.0 | 24 55.9 ± 1.3 |
| Whole Blood | 33 72.1 ± 0.4 | 36 28.4 ± 0.2 | 34 38.1 ± 0.9 | 26 54.7 ± 1.7 |
| Testis | 34 71.0 ± 0.1 | 42 27.4 ± 0.2 | 39 35.8 ± 1.2 | 33 52.0 ± 0.2 |
| Uterus Corpus | 35 70.8 ± 2.0 | 5 34.2 ± 0.2 | 21 42.3 ± 1.1 | 41 48.9 ± 3.3 |
| Tongue | 36 70.1 ± 0.2 | 12 33.2 ± 0.7 | 29 39.4 ± 1.1 | 40 49.0 ± 0.4 |
| Testis Seminiferous Tubule | 37 69.5 ± 0.1 | 47 26.1 ± 0.1 | 46 33.8 ± 0.2 | 35 50.8 ± 0.1 |
| Skeletal Muscle | 38 69.1 ± 0.1 | 34 29.3 ± 0.1 | 36 36.6 ± 0.1 | 36 49.9 ± 0.1 |
| Testis Interstitial | 39 68.7 ± 0.3 | 49 25.3 ± 0.2 | 47 33.6 ± 0.2 | 38 49.2 ± 0.3 |
| Ciliary Ganglion | 40 68.42 ± 0.04 | 37 28.4 ± 0.1 | 42 35.44 ± 0.04 | 37 49.3 ± 0.1 |
| Trachea | 41 68.3 ± 0.8 | 43 27.3 ± 1.7 | 35 37.9 ± 1.3 | 49 46.5 ± 1.5 |
| Superior Cervical Ganglion | 42 68.3 ± 0.1 | 38 28.21 ± 0.01 | 38 35.8 ± 0.1 | 39 49.17 ± 0.03 |
| Dorsal Root Ganglion | 43 67.8 ± 0.1 | 45 27.0 ± 0.1 | 40 35.6 ± 0.1 | 43 48.5 ± 0.1 |
| Whole Genome | 44 67.79 ± 0.01 | 33 29.38 ± 0.03 | 43 35.031 ± 0.004 | 44 48.23 ± 0.01 |
| Trigeminal Ganglion | 45 67.53 ± 0.03 | 41 27.9 ± 0.1 | 41 35.5 ± 0.1 | 42 48.82 ± 0.01 |
| Skin | 46 67.5 ± 0.2 | 44 27.1 ± 0.5 | 45 34.4 ± 0.7 | 47 47.1 ± 0.4 |
| Atrioventricular Node | 47 67.401 ± 0.005 | 40 27.93 ± 0.01 | 44 35.0 ± 0.1 | 45 48.21 ± 0.01 |
| Lymph Node | 48 65.3 ± 1.1 | 46 27.0 ± 2.0 | 32 38.5 ± 3.4 | 48 47.0 ± 2.0 |
| Fetal Liver | 49 64.0 ± 0.3 | 48 25.9 ± 0.9 | 50 28.8 ± 1.3 | 46 47.4 ± 0.7 |
| Appendix | 50 63.17 ± 0.05 | 50 24.1 ± 0.1 | 49 30.5 ± 0.4 | 50 43.8 ± 0.8 |

* Ranking of G4 enrichment in TRRs of genes active in a specific tissue was performed by comparing the mean value of G4 enrichment in that tissue with other tissues.
