# Supplementary material for: Possible Regulatory Roles of Promoter G-Quadruplexes in Cardiac Function-Related Genes – Human TnIc as a Model
Source: PLoS One. 2013 Jan 9;8(1):e53137. doi: 10.1371/journal.pone.0053137 (PMC3541360; doi:10.1371/journal.pone.0053137)
Supplement: Table S2 — CF o and CQ o values of TRRs of genes containing well-studied promoter G4s. (DOC) [file pone.0053137.s012.doc]

**Table S2.** *CF*_o_ and *CQ*_o_ values of TRRs of genes containing well-studied promoter G4s

| **Gene Name** | **Ensembl ID** | ***CF*_o_ (%)** | ***CQ*_o_ (%)** |
| --- | --- | --- | --- |
| *BCL-2* | ENSG00000171791 | 50.1 | 56.1 |
| *KIT* | ENSG00000157404 | 43.5 | 73.4 |
| *MYC* | ENSG00000136997 | 80.6 | 64.1 |
| *HIF1-α* | ENSG00000100644 | 55.1 | 56.6 |
| *MYB* | ENSG00000118513 | 82.9 | 77.5 |
| *PDGF-A* | ENSG00000197461 | 96.2 | 78.0 |
| *PDGFR-β* | ENSG00000113721 | 72.2 | 63.9 |
| *TERT* | ENSG00000164362 | 68.7 | 52.9 |
| *VEGF-A* | ENSG00000112715 | 75.3 | 68.9 |
