# Supplementary material for: Possible Regulatory Roles of Promoter G-Quadruplexes in Cardiac Function-Related Genes – Human TnIc as a Model
Source: PLoS One. 2013 Jan 9;8(1):e53137. doi: 10.1371/journal.pone.0053137 (PMC3541360; doi:10.1371/journal.pone.0053137)
Supplement: Table S3 — Parameters obtained from curve fittings of TnIc MNSG4 and −80 G4 unfolding in solution. (DOC) [file pone.0053137.s013.doc]

**Table S3. Parameters obtained from curve fittings of TnIc MNSG4 and -80G4 unfolding in solution.**

| **Temperature / °C** | **<*τ*> / s** | ***k* / 10^-5^s^-1^** | ***E*_a_ / kJ mol^-1^** | **Δ*H*^‡^ / kJ mol^-1^** | **Δ*S*^‡^ / J mol^-1^** |
| --- | --- | --- | --- | --- | --- |
| ***TnIc MNSG4 (fast decaying component)*** | | | | | |
| 25 | 126.7 ± 6.2 | 789.3 ± 38.6 | -- | -- | -- |
| 35 | 128.5 ± 3.4 | 778.2 ± 20.6 |
| 45 | 127.4 ± 2.2 | 784.9 ± 13.6 |
| 55 | 140.2 ± 1.6 | 713.3 ± 8.1 |
| ***TnIc MNSG4 (slow decaying component)*** | | | | | |
| 25 | 4,438.0 ± 32.1 | 22.5 ± 0.2 | 22.1 ± 0.4 | 19.6 ± 0.4 | -249.2 ± 1.2 |
| 35 | 3,388.9 ± 16.3 | 29.5 ± 0.1 |
| 45 | 2,538.9 ± 16.2 | 39.4 ± 0.3 |
| 55 | 1,969.5 ± 11.1 | 50.8 ± 0.3 |
| ***TnIc -80G4-F (fast decaying component)*** | | | | | |
| 35 | 3,366.6 ± 46.7 | 29.7 ± 0.4 | 86.1 ± 23.7 | 83.4 ± 23.6 | -44.1 ± 7.4 |
| 45 | 1,669.2 ± 18.9 | 59.9 ± 0.7 |
| 55 | 1,094.0 ± 13.6 | 91.4 ± 1.1 |
| 65 | 136.5 ± 3.4 | 732.1 ± 18.4 |
| ***TnIc -80G4-S (slow decaying component)*** | | | | | |
| 35 | 173,840.9 ± 5,542.6 | 0.6 ± 0.1 | 100.6 ± 6.6 | 98.0 ± 6.5 | -27.1 ± 20.0 |
| 45 | 44,424.4 ± 482.3 | 2.3 ± 0.1 |
| 55 | 18,811.2 ± 136.0 | 5.3 ± 0.1 |
| 65 | 4,789.9 ± 32.8 | 20.9 ± 0.2 |

G4 unfolding was measured by the decrease of Cy5 fluorescence when hybridised by C-rich complimentary strand. Experiments were carried out in solution containing 10 mM Tris-HCl (pH 7.4) and 100 mM K^+^ at different temperatures.
