# Supplementary material for: Possible Regulatory Roles of Promoter G-Quadruplexes in Cardiac Function-Related Genes – Human TnIc as a Model
Source: PLoS One. 2013 Jan 9;8(1):e53137. doi: 10.1371/journal.pone.0053137 (PMC3541360; doi:10.1371/journal.pone.0053137)
Supplement: Protocols S1 — Supporting protocols including G4-binding ligand synthesis, Electrophoretic mobility shift assay, CD characterization, DMS footprinting, unfolding kinetics and the human genome sequence data bases used in the bioinformatic analysis. (DOC) [file pone.0053137.s014.doc]

**Supporting Information**

**Supporting Protocols**

**G4-binding ligand synthesis**

The platinum complex **1** and its precursor [Pt(terpy-O-morph)Cl]Cl, as well as the di-platinum and di-copper complexes **2** and **3**, were synthesized following the protocol we recently reported [1,2]. Structures of these complexes are shown in Figure 7a.

**Synthesis of [Pt(terpy-O-morph)(S-pip)](PF_6_)_3_**

[Pt(terpy-O-morph)Cl]Cl (100 mg, 0.16 mmol) and *N*-(2-sulfanylethyl)piperidine (139 mg, 0.96 mmol) were stirred in water (15 ml) at 40^o^C for 48 h to afford an orange solution. After removing excess thiol by extraction with diethyl ether (20 ml × 3) the aqueous layer was removed under reduced pressure. The resultant solid was dissolved in the minimum amount of DMSO, to which excess NaPF_6_ (as an aqueous solution) was added dropwise. This yielded a red precipitate which was isolated by filtration and washed repeatedly with diethyl ether. The compound was isolated as an orange powder (23 mg, 21%); ^1^H NMR (400 MHz, DMSO-d_6_): δ_H_ 9.39 (d, 2H, ^3^*J*_HH_ 4.0, tpy 2-H), 8.70 (d, 2H, ^3^*J*_HH_ 6.0, tpy 5-H), 8.57 (t, 2H, ^3^*J*_HH_ 6.0, tpy 4-H), 8.41 (s, 2H, tpy 3’ & 5’-H), 8.01 (t, 2H, ^3^*J*_HH_ 6.0, tpy 3-H), 4.70 (br s, 2H, O-ethyl 1-H), 4.07 (br s, 2H, O-ethyl 2-H), 3.76 (br s, 4H, O-morpholine, 1-H), 3.60 (br s, 4H, O-morpholine, 2-H), 3.09 (t, 2H, ^3^*J*_HH_ 8.0, S-ethyl 1-H), 2.95 (t, 2H, ^3^*J*_HH_ 8.0, S-ethyl 2-H), 2.82 (m, 4H, S-piperidine, 1-H), 1.78 (m, 4H, S-piperidine, 2-H), 1.61 (m, 2H, piperidine N^+^-H and morpholine N^+^-H), 1.33 (m, 2H, S-piperidine 3-H); ^31^P NMR (400MHz, DMSO-d_6_): δ_P_ -144.20 (sept, 3P, ^1^*J*_PF_ 1752, PF_6_);ESI-MS Calcd. C_28_H_38_F_18_N_5_O_2_P_3_PtS (M^+^): 1136.1 a.m.u. Found (M-1PF_6_^+^): 993.2 a.m.u., (M-2PF_6_^+^): 847.2 a.m.u., (M-3PF_6_^+^): 701.2 a.m.u.; Anal. Calcd. For C_28_H_38_F_18_N_5_O_2_P_3_PtS∙3H_2_O: C 28.20, H 3.72, N 5.87 Found: C 28.02, H 3.44, N 5.50.

**Electrophoretic mobility shift assay (EMSA)**

G4-forming and non-G4-forming DNA oligos were dissolved in 10 mM Tris-HCl buffer (pH 7.4) with 100 mM KCl, and diluted to 5 μM. Prior to native EMSA experiments, G4-forming oligos were incubated at 95 °C for 15 min, then gradually cooled down to room temperature over 4 hours. Oligos were then loaded to 12 % native polyacrylamide gel (PAGE) in 1xTris-borate-EDTA (TBE) buffer for electrophoresis. In control experiments, oligos were incubated at 95 °C for 15 min, and then left on ice before loading to denaturing PAGE containing 7 M urea. All EMSA experiments were carried out at 90 V at room temperature.

**CD characterization and CD melting**

The test G4-binding compounds were dissolved in DMSO (dimethyl sulfoxide) to yield a 1 mM stock solution. This was then diluted using 10 mM Tris-HCl buffer (10 mM, pH 7.4) supplemented with 100 mM cation ions to the appropriate concentrations. DNA oligo samples in the absence of G4-binding compounds were diluted to 5 μM in Tris-HCl buffer (10 mM, pH 7.4) with 100 mM KCl, NaCl or LiCl. Prior to CD measurements, the DNA oligo samples were annealed by heating to 95 °C for 15 minutes and then by cooling to room temperature over 4 hours. To test the stabilising ability of G4-binding compounds, DNA oligo samples at 5 μM concentration were mixed with 10 μM compounds, and annealed by following the same protocol. CD spectra were recorded on an Chirascan spectrometer (Applied Photophysics Ltd., Surrey, UK) at 25 °C with following parameters: bandwidth- 1 nm, spectral range- 230 ~ 330 nm, step size- 0.5 nm, and time per point- 1 second. 1 mm optical path length quartz cell was used. The baselines of all CD spectra were corrected by using blank buffer. The CD spectra were collected and processed using the Chirascan and Chirascan Viewer softwares (Applied Photophysics Ltd.) respectively. CD melting spectra were recorded at 2 °C intervals while the temperature was increased at a rate of 2 °C/min with 2 minutes dwelling time for each temperature. All melting curves were analysed in Origin 7.0 (OriginLab Corporation, Northampton, MA, USA) by following a simple two-state transition model proposed previously [3].

**DMS footprinting**

Oligos **Tr_MNS_-I** and **Tr_-80_-I** were 5’-end-labeled with [γ-^32^P]ATP (Perkin Elmer, Cambridge, UK) using T4 polynucleotide kinase (NEB, Hitchin, UK) for 1 hour at 37 °C. After inactivating the labelling reaction at 95 °C for 15 min, the 5’-end-labeled oligos were purified by 12 % denaturing PAGE containing 7 M urea. Purified oligos were incubated as stated above to allow formation of G4s in 10 mM Tris-HCl (pH 7.4) buffer with 100 mM KCl. Intramolecular G4s from oligo **Tr_MNS_-I** and **Tr_-80_-I** were then separated by 12 % native PAGE. DMS footprinting was carried out as previously described [4]. Generally, 35 000 cpm purified intramolecular G4s were diluted with 0.1 x TE buffer to 90 μl per reaction. After adding 1 μl of calf thymus DNA (0.1 μg/μl), DMS methylation reaction was carried out for 5 min by adding 1 μl 25 % DMS solution (1:4 DMS/ethanol vol/vol). Each reaction was stopped by adding 18 μl stop buffer (1:6:7 β-mercaptoethanol/water/NaOAc, vol/vol (3M NaOAc)), and methylated oligos were recovered by ethanol precipitation. Piperidine cleavage was performed in 30 μl piperidine at 90 °C for 30 min. Cleavage products were air dried and run in a 20 % sequencing gel. Dried gel was imaged by phosphor screen (GE Healthcare, Hatfield, UK).

**Unfolding kinetics**

For both MNSG4 and -80G4 systems, their unfolding kinetics were investigated via a pseudo-first order approach. Generally, 100 nM Cy3-labeled MNSG4 (oligo **Tr_MNS_-III**) and -80G4 (oligo **Tr_-80_-III**) were mixed with 150 nM Cy5-labeled 3’ linker (oligo **Comp-Cy5**) in 10 mM Tris-HCl buffer containing 100 mM K^+^. Both G4 samples were annealed to allow the formation G4 as stated previously. Fluorescence signals of 200 μl annealed G4 samples at 100 nM concentration were measured by Fluorometer (HORIBA, Kyoto, Japan). Samples were excited at 550 nm, and Cy5 fluorescence signals were collected at 664 nm with 5 nm slit (peak of Cy5 emission). Cy5 signals were measured at 1 second time interval with 0.5 second time bin. Intensity of Cy5 fluorescence signals in the absence of C-rich oligos was monitored for 30 minutes prior to each kinetics experiment. If the signal was found to be systematically changing, it was allowed to further stabilise. 4 μl DNA solution containing 100 μM C-rich complimentary strand (oligo **Tr_MNS_-II** for MNSG4, and oligo **Tr_-80_-II** for -80G4) was mixed with 200 μl G4 samples (100 nM oligo, 10 mM Tris-HCl, and 100 mM K^+^) to give a final concentration of 2 μM at the time 0 of the unfolding reaction. The progress of G4 unfolding was monitored by following the decrease in Cy5 fluorescence as the duplex formed. Unfolding kinetics runs of single repeat of MNSG4 and -80G4 were repeated at different temperatures (MNSG4: 25, 35, 45, and 55 °C, and -80G4: 35, 45, 55 and 65 °C). In each measurement, the mixing time (adding C-rich complimentary oligo to the G4 samples) was controlled within 10 seconds, and hence has only little influence to determine unfolding kinetic parameter accurately. The cuvette was sealed by a cap in the measurement to prevent evaporation at higher temperature. The resulting traces of Cy5 fluorescence were fitted by a double exponential decay model in Origin 7.0.

**The human genome sequences**

Gzip compressed sequences of the human genome (version 37.64) were downloaded from ENSEMBL FTP (<ftp://ftp.ensembl.org/pub/release-65/fasta/homo_sapiens/dna/>) with file names as Homo_sapiens.GRCh37.64.dna.chromosome.*.fa.gz (* indicates chromosome number). Gzip compressed sequence files of the human genome were then decompressed to fasta format.

**Overall gene/transcript features**

Gene/transcript features including ENSEMBL gene ID, ENSEMBL transcript ID, chromosome number, strand polarity, gene start/end position, transcript start/end position, gene description, EntrezGene ID, transcript biotype and gene biotype were exported to a csv format file (comma-separated values) from BioMart (<http://www.biomart.org/biomart/martview/>). When exporting gene features, “Gene type” and “Status” of genes and transcripts were selected as “protein_coding” and “known”, respectively.

**Tissue-specific gene/transcript features**

For tissue-specific G4 enrichment analysis, tissue-specific gene/transcript lists were exported in csv format by setting “GNF/Atlas organism part” to different tissues in the BioMart. Gene/transcript lists active in 60 different tissues (tissue-specific genes) and one list including all gene/transcript (at whole genome level) were exported, while 11 of them with transcripts number ≤ 800 (i.e. low abundance transcripts) were excluded in subsequent analysis due to limited gene/transcript available (salivary gland, pancreas, tonsil, beta cell islets, lymph, fetal lung, pituitary, thymus, uterus, ovary, and adrenal cortex).

**Pathway-specific gene/transcript features**

A csv format file containing component genes for all human pathways (234 pathways) was downloaded from KEGG (Kyoto Encyclopedia of Genes and Genomes) PATHWAY database (<http://www.genome.jp/kegg/kegg2.html>). In each pathway, KEGG ID of each gene was mapped to its ENSEMBL gene ID, and gene features were obtained according to the previously exported csv file from BioMart. All pathways including more than 50 genes were subjected to subsequent pathway analysis. Transcriptional regulatory regions (TRRs) of individual transcripts were defined as -2,000 bp to +1,000 bp from TSS of the transcript. Coding strand sequences of TRRs were extracted from the human genome sequence files, according to the transcript features (i.e. chromosome number, strand polarity, and transcript start site). G4-forming motifs on both strands were defined as G_≥3_(N_1~7_G_≥3_)_≥3_.

**Redundancy issue**

In the csv file showing gene/transcript features, most genes are composed of multiple transcripts, which would induce bias in subsequent analysis. Thus, in this study, to take into account this redundancy issue, only one transcript was randomly chosen to represent one gene, and the whole analysis was repeated for 10 times. Briefly, in tissue-specific and pathway-specific analysis, transcripts representing the same gene (with the same ENSEMBL gene ID) were grouped together, and one transcript was chosen according to a uniformly distributed pseudorandom number generated by MATLAB 2009 (MathWorks) using the built-in function “unidrnd”. In each iteration, a single transcript representing a single gene without redundancy was randomly chosen, and the result was presented as mean ± standard deviation (SD) from 10 iterations. Differences between iterations were evaluated by one-way ANOVA test, in which *P* ≥ 0.05 represents no significant difference was found.

**Supporting References**

1. Suntharalingam K, White AJ, Vilar R (2010) Two metals are better than one: investigations on the interactions between dinuclear metal complexes and quadruplex DNA. Inorg Chem 49: 8371-8380.

2. Suntharalingam K, White AJ, Vilar R (2009) Synthesis, structural characterization, and quadruplex DNA binding studies of platinum(II)-terpyridine complexes. Inorg Chem 48: 9427-9435.

3. Greenfield NJ (2006) Using circular dichroism collected as a function of temperature to determine the thermodynamics of protein unfolding and binding interactions. Nat Protoc 1: 2527-2535.

4. Siddiqui-Jain A, Grand CL, Bearss DJ, Hurley LH (2002) Direct evidence for a G-quadruplex in a promoter region and its targeting with a small molecule to repress c-MYC transcription. Proc Natl Acad Sci U S A 99: 11593-11598.
